# Building Programs to Eradicate Toxoplasmosis Part IV: Understanding and Development of Public Health Strategies and Advances “Take a Village”

**DOI:** 10.1007/s40124-022-00268-x

**Published:** 2022-08-16

**Authors:** Mariangela Soberón Felín, Kanix Wang, Aliya Moreira, Andrew Grose, Karen Leahy, Ying Zhou, Fatima Alibana Clouser, Maryam Siddiqui, Nicole Leong, Perpetua Goodall, Morgan Michalowski, Mahmoud Ismail, Monica Christmas, Stephen Schrantz, Zuleima Caballero, Ximena Norero, Dora Estripeaut, David Ellis, Catalina Raggi, Catherine Castro, Davina Moossazadeh, Margarita Ramirez, Abhinav Pandey, Kevin Ashi, Samantha Dovgin, Ashtyn Dixon, Xuan Li, Ian Begeman, Sharon Heichman, Joseph Lykins, Delba Villalobos-Cerrud, Lorena Fabrega, José Luis Sanchez Montalvo, Connie Mendivil, Mario R. Quijada, Silvia Fernández-Pirla, Valli de La Guardia, Digna Wong, Mayrene Ladrón de Guevara, Carlos Flores, Jovanna Borace, Anabel García, Natividad Caballero, Claudia Rengifo-Herrera, Maria Theresa Moreno de Saez, Michael Politis, Stephanie Ross, Mimansa Dogra, Vishan Dhamsania, Nicholas Graves, Marci Kirchberg, Kopal Mathur, Ashley Aue, Carlos M. Restrepo, Alejandro Llanes, German Guzman, Arturo Rebellon, Kenneth Boyer, Peter Heydemann, A. Gwendolyn Noble, Charles Swisher, Peter Rabiah, Shawn Withers, Teri Hull, David Frim, David McLone, Chunlei Su, Michael Blair, Paul Latkany, Ernest Mui, Daniel Vitor Vasconcelos-Santos, Alcibiades Villareal, Ambar Perez, Carlos Andrés Naranjo Galvis, Mónica Vargas Montes, Nestor Ivan Cardona Perez, Morgan Ramirez, Cy Chittenden, Edward Wang, Laura Lorena Garcia-López, Guillermo Padrieu, Juliana Muñoz-Ortiz, Nicolás Rivera-Valdivia, María Cristina Bohorquez-Granados, Gabriela Castaño de-la-Torre, Juan David Valencia Hernandez, Daniel Celis-Giraldo, Juan Alejandro Acosta Dávila, Elizabeth Torres, Manuela Mejia Oquendo, José Y. Arteaga-Rivera, Dan L Nicolae, Andrey Rzhetsky, Nancy Roizen, Eileen Stillwaggon, Larry Sawers, Francois Peyron, Martine Wallon, Emanuelle Chapey, Pauline Levigne, Carmen Charter, Migdalia De Frias, Jose Montoya, Cindy Press, Raymund Ramirez, Despina Contopoulos-Ioannidis, Yvonne Maldonado, Oliver Liesenfeld, Carlos Gomez, Kelsey Wheeler, Samantha Zehar, James McAuley, Denis Limonne, Sandrine Houze, Sylvie Abraham, Raphael Piarroux, Vera Tesic, Kathleen Beavis, Ana Abeleda, Mari Sautter, Bouchra El Mansouri, Adlaoui El Bachir, Fatima Amarir, Kamal El Bissati, Ellen Holfels, David Frim, David McLone, Richard Penn, William Cohen, Alejandra de-la-Torre, Gabrielle Britton, Jorge Motta, Eduardo Ortega-Barria, Isabel Luz Romero, Paul Meier, Michael Grigg, Jorge Gómez-Marín, Jagannatha Rao Kosagisharaf, Xavier Sáez Llorens, Osvaldo Reyes, Rima McLeod

**Affiliations:** 1 Toxoplasmosis Programs and Initiatives in Panama, Ciudad de Panama, Panama; 2 Institute for Genomics and Systems Biology, The University of Chicago, Chicago, IL USA; 3 Pritzker School of Medicine, The University of Chicago, Chicago, IL USA; 4 Instituto de Investigaciones Científicas y Servicios de Alta Tecnología AIP (INDICASAT-AIP), Ciudad de Panama, Panama; 5 Department of Pediatrics Infectious Diseases/Department of Neonatology, Hospital del Niño doctor José Renán Esquivel, Ciudad de Panama, Panama; 6 Department of Ophthalmology and Visual Sciences, The University of Chicago, Chicago, IL USA; 7 The College, The University of Chicago, Chicago, IL USA; 8 The Global Health Center, The University of Chicago, Chicago, IL USA; 9 Department of Statistics, The University of Chicago, Chicago, IL USA; 10 Rush University Medical School/Rush University Medical Center, Chicago, IL USA; 11 Academia Interamericana de Panama, Ciudad de Panama, Panama; 12 Hospital Santo Tomás, Ciudad de Panama, Panama; 13 Hospital San Miguel Arcángel, Ciudad de Panama, Panama; 14 Universidad de Panama, Ciudad de Panama, Panama; 15 Global Health Center Capstone Program, The University of Chicago, Chicago, IL USA; 16 Harris School of Public Policy, The University of Chicago, Chicago, IL USA; 17 Sanofi Aventis de Panama S.A., University of South Florida, Ciudad de Panama, Panama; 18 Northwestern University Feinberg School of Medicine, Chicago, IL USA; 19 NorthShore Evanston Hospital, Evanston, IL USA; 20 Department of Microbiology, The University of Tennessee, Knoxville, TN USA; 21 Universidad de Federal de Minas Gerais, Minas Gerais, Brazil; 22 Universidad Autónoma de Manizales, Manizales, Colombia; 23 Universidad del Quindío, Armenia, Colombia; 24 The University of South Florida College of Public Health, Tampa, FL USA; 25 Grupo de Investigación en Neurociencias, Universidad del Rosario, Bogotá, Colombia; 26 Department of Economics, Gettysburg College, Gettysburg, PA USA; 27 Department of Economics, American University, Washington, DC USA; 28 Institut des agents infectieux, Hôpital de la Croix-Rousse, Lyon, France; 29 Remington Specialty Laboratory, Palo Alto, CA USA; 30 Department of Pediatrics, Division of Infectious Diseases, Stanford University College of Medicine, Stanford, CA USA; 31 Roche Molecular Diagnostics, Pleasanton, CA USA; 32 LDBioDiagnostics, Lyon, France; 33 Laboratory of Parasitologie, Bichat-Claude Bernard Hopital, Paris, France; 34 INH, Rabat, Morocco; 35 Faculty of Sciences Ain Chock, University Hassan II, Casablanca, Morocco; 36 Sistema Nacional de investigadores de Panama (SNI), Panama, Panama; 37 Secretaría Nacional de Ciencia, Tecnología e Innovación (SENACYT), Ciudad de Panama, Panama; 38 GSK Vaccines, Panama, Panama; 39 Molecular Parasitology, NIAID,NIH, Bethesda, MD USA; 40 Toxoplasmosis Center, The University of Chicago and Toxoplasmosis Research Institute, Chicago, IL USA; 41 Department of Pediatrics (Infectious Diseases), The University of Chicago, Chicago, IL USA

**Keywords:** congenital toxoplasmosis, diagnostic testing, prenatal screening, point-of-care testing, antiparasitic treatment, marginalized populations

## Abstract

**Purpose of Review:**

Review international efforts to build a global public health initiative focused on toxoplasmosis with spillover benefits to save lives, sight, cognition and motor function benefiting maternal and child health.

**Recent Findings:**

Multiple countries’ efforts to eliminate toxoplasmosis demonstrate progress and context for this review and new work.

**Summary:**

Problems with potential solutions proposed include accessibility of accurate, inexpensive diagnostic testing, pre-natal screening and facilitating tools, missed and delayed neonatal diagnosis, restricted access, high costs, delays in obtaining medicines emergently, delayed insurance pre-approvals and high medicare copays taking considerable physician time and effort, harmful shortcuts being taken in methods to prepare medicines in settings where access is restricted, reluctance to perform ventriculoperitoneal shunts promptly when needed without recognition of potential benefit, access to resources for care, especially for marginalized populations, and limited use of recent advances in management of neurologic and retinal disease which can lead to good outcomes.

**Supplementary Information:**

The online version contains supplementary material available at 10.1007/s40124-022-00268-x.

## Introduction

The earlier papers in this series presented studies by students and colleagues focused on educational tools and their efficacy in Panamá’, Colombia and the United States and on epidemiological aspects of *Toxoplasma* infection and toxoplasmosis in the USA, Europe, Morocco, Brazil, Panamá’ and Colombia. It is clear from earlier studies in the United States [1••, 2, 3••, 4••, 5••, 6••, 7••, 8••, 9••, 10••, 11••, 12, 13, 14••, 15••, 16••, 17••, 18•, 19•, 20•, 21•, 22•, 23, 24••, 25, 26, 27•, 28•, 29–30, 31•, 32•, 33••, 34•, 35•, 36, 37•, 38•, 39••, 40•, 41•, 42••, 43••, 44••, 45, 46•, 47••, 48••, 49, 50•], and the present studies that access to inexpensive, effective diagnostic tools and anti-parasitic treatment( and ultimately vaccines) [1••, 2, 3••, 4••, 5••, 6••, 7••, 8••, 9••, 10••, 11••, 12, 13, 14••, 15••, 16••, 17••, 18•, 19•, 20•, 21•, 22•, 23, 24••, 25, 26, 27•, 28•, 29–30, 31•, 32•, 33••, 34•, 35•, 36, 37•, 38•, 39••, 40•, 41•, 42••, 43••, 44••, 45, 46•, 47••, 48••, 49, 50•, 51•, 52, 53••, 54, 55••, 56••, 57•, 58••, 59•, 60••, 61, 62••, 63, 64.••••, 65, 66••, 67••, 68••, 69, 70, 71.••••, 72••, 73••, 74••, 75••, 76–78, 79••, 80••, 81••, 82, 83, 84••, 85••, 86••, 87••, 88••, 89••, 90, 91, 92••, 93, 94, 95, 96, 97, 98••, 99••, 100, 101••, 102••, 103••, 104••, 105••, 106••, 107••, 108••, 109••, 110, 111, 112, 113••, 114••, 115••, 116••, 117••, 118••, 119••, 120, 121, 122••, 123••, 124, 125••, 126••, 127••, 128••, 129••, 130••, 131••, 132, 133••, 134••, 135, 136••] are/will be critical to prevent this infection and its adverse sequelae. This is especially true for marginalized populations. This fourth paper reviews, considers and discusses results in the first three manuscripts and reviews and presents updates with additional data about solving ongoing problems. These are reviewed and summarized in order to emphasize the importance of, and steps to build, programs to address the clinical and public health challenges of preventing congenital toxoplasmosis. This analysis and discussion places work in the United States [1••, 2, 3••, 4••, 5••, 6••, 7••, 8••, 9••, 10••, 11••, 12, 13, 14••, 15••, 16••, 17••, 18•, 19•, 20•, 21•, 22•, 23, 24••, 25, 26, 27•, 28•, 29–30, 31•, 32•, 33••, 34•, 35•, 36, 37•, 38•, 39••, 40•, 41•, 42••, 43••, 44••, 45, 46•, 47••, 48••, 49, 50, 52, 55••, 56••], Panama [59•] and Colombia [63, 65, 97], in the context of progress in other countries [51•, 52, 53••, 54, 55••, 56••, 57•, 58••, 59•, 60••, 61, 62••, 63, 64.••••, 65, 66••, 67••, 68••, 74••, 75••, 77, 92••, 100]. For the United States, in Part 1 there was a focus primarily on the work in the U.S. National Collaborative Chicago-based Congenital Toxoplasmosis Study ( NCCCTS, 1981 to present) as a whole with brief mention of and/or reference to some of the other US and European, as well as Brazilian studies and work [1••, 2, 3••, 4••, 5••, 6••, 7••, 8••, 9••, 10••, 11••, 12, 13, 14••, 15••, 16••, 17••, 18•, 19•, 20•, 21•, 22•, 23, 24••, 25, 26, 27•, 28•, 29–30, 31•, 32•, 33••, 34•, 35•, 36, 37•, 38•, 39••, 40•, 41•, 42••, 43••, 44••, 45, 46•, 47••, 48••, 49, 50•, 52, 55••, 57•, 58••, 68••, 76, 88••, 89••, 90, 91, 92••, 93, 94, 95, 116••, 117••].

Part IV considers briefly the foundation to which these studies contribute, providing a rationale and framework for extending the studies. This includes additional basic science and translational work and needed improvements in clinical care [1••, 2, 3••, 4••, 5••, 6••, 7••, 8••, 9••, 10••, 11••, 12, 13, 14••, 15••, 16••, 17••, 18•, 19•, 20•, 21•, 22•, 23, 24••, 25, 26, 27•, 28•, 29–30, 31•, 32•, 33••, 34•, 35•, 36, 37•, 38•, 39••, 40•, 41•, 42••, 43••, 44••, 45, 46•, 47••, 48••, 49, 50•, 51•, 52, 53••, 54, 55••, 56••, 57•, 58••, 64.••••, 68••, 88••, 89••, 90, 91, 92••, 93, 94, 118••, 124, 125••, 126••, 127••] and potential novel treatments and vaccines [84••, 85••, 86••, 87••, 88••, 98••, 99••, 100, 101••, 102••, 103••, 104••, 105••, 106••, 107••, 108••, 109••, 110, 111, 112, 113••, 114••, 115••, 116••, 117••, 118••, 119••, 120, 121, 122••, 123••, 124, 128••, 129••, 131••, 132, 133••, 134••, 135, 136••, 137••, 138••, 139••] including vaccines to prevent oocyst shedding by cats [95, 96]. Some of this work is ongoing in the Toxoplasmosis Center in Chicago, in other programs in the US and in other countries.

## Approach

### Overview and Summary

Initially, we consider the clinical manifestations and the benefits from optimizing clinical care pre- and postnatally. We compare approaches in several countries, individually considering strengths, and where problems have arisen/arise and present possible solutions. This analysis addresses available approaches, a ^«^ toolbox ^»^ of recent advances that can contribute to eliminating toxoplasmosis through optimizing care and preventable and therapeutic approaches. This includes a novel “toolbox” of diagnostic tools and potential preventative and therapeutic approaches. These developments and approaches are summarized in Tables 1, 2, 3, 4 and 5. These solutions have involved/ will involve a variety of skill sets and persons, « taking :a village » of many physicians, scientists, students and others in an ongoing global initiative.

**Table 1:** Development of understanding of need for a WHO ASSURED * Criteria point-of-care testing functional access to correct medicines to initiate medicines immediately and promptly for this medical emergency in this disease in the US

Date	Source of information	Information
1950–1981	France, Austria	Treatment appears beneficial, the earlier the better; laws passed in France and Austria mandating prenatal screening.
1981–1990	France, Austria, United States	Possible to treat continuously with pyrimethamine and sulfadiazine, Phase 1 clinical trial.
1990–2008	United States	Phase 2 and later phase trials.
2010	Illinois, United States	Attempt to introduce a legislation in Springfield, need for infrastructure, inexpensive test materials, and medicines. Identified cost-benefit macro-algorithm, spillover benefit identified, inexpensive testing tools identified.
2014	Worldwide	Global initiative begun.
2015	United States	Price of pyrimethamine increased from $0.01 ner pill to $750 per pill. **
2021	France, United States, Morocco, Colombia, Panama	Improved inexpensive tests, meeting WHO/assured criteria, CE-marked criteria, FDA clearance pending.

*Affordability, Sensitivity, User-friendly, Rapid and robust, Equipment-free and Deliverable to those who need it

**Cost of medicines and import restrictions have caused limitations in use in the US and Panamá. The problems with cost and access discussed in Part I of the manuscripts in this series may be resolving if the source material is of high quality and reliability. This is a new development in early 2022 and details are not yet available

**Table 2.**
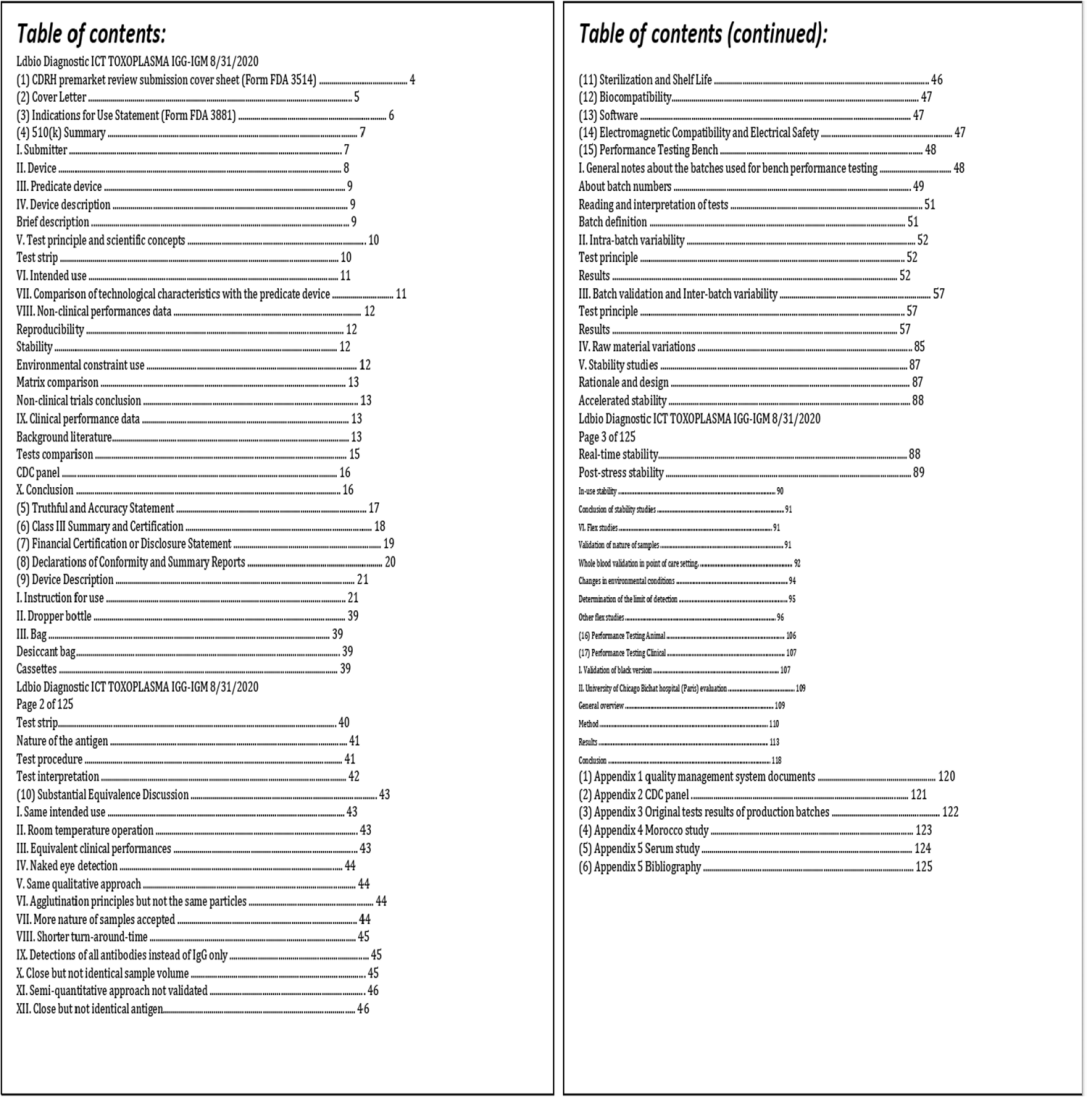
Table contents for materials prepared for FDA review of 510k and CLIA waiver

**Table 3:** Development of point-of-care (POC) and a revolutionary nearly 100% sensitive and specific test for acquisition of *Toxoplasma gondii*

Concept	References
Development of test	[80••]
Application of test to serum	[43••]
Applicability to infections in the United States	[43••]
Meeting WHO ASSURED criteria with POC test	[44••]
Implementation and building a global initiative	[33••, 39••, 48••, 52, 55••, 58••, 60••, 61, 66••, 67••, 81••]
Scaling in Morocco, Panamá, Colombia, Brazil, Argentina, Europe, and the U.S.	[57•], In Progress
Multiplexing: Nano-Gold test	[82]

**Table 4:** Summary of evaluation performed using LDBIO Toxoplasma ICT IgG-IgM test

Study	Published?	Sample	Npos	Nneg	Se	Sp	Comparative method
Black variant validation	No	Serum	483	291	98.5%	99.7%	CRS
El Mansouri, 2021	Yes	Whole blood	226	406	97%	100%	CRS
Bichat/Chicago^a^	Ongoing	Serum	169	1215	98.2%^a^	99.8%^a^	CRS
Chapey, 2016^b^	Yes	Serum	109	291	97%	96%	Architect
Mahinc, 2017^b^	Yes	Serum	339	663	100%	98.7%	CRS
Begeman, 2017^b^	Yes	Serum	129	51	100%	100%	CRS
Lykins, 2018	Yes	Whole Blood	101	143	100%	100%	CRS
University of Chicago	No	Whole Blood	5	35	100%	100%	Bioplex ToRC
Gomez, 2018^c^	Yes	Serum	170	140	100%^c^	98.8%^c^	CRS^c^

^a^As the study is still under redaction, published performances may differ

^b^Former pink variant CRS: composite reference standard, using more than a single test to determine status. See methodology of each study for details concerning the CRS used

^c^including CDC100 panel (100% Se/Sp)

*Abbreviations*: *N* number, *pos* presence of *T.gondii* specific antibody, *neg* absence of antibody to *T.gondii*, *Se* sensitivity, *Sp* specificity

**Table 5.** Development of novel, improved medicines and vaccines to prevent, cure and eliminate *Toxoplasma* infection

	Approach to elimination with medicines, vaccines, environment approaches	References (Selected examples)
Medicines	Novel use of established medicines	[19•]
	New approaches to eliminating toxoplasma by small molecules and antisense	[85••, 86••, 87]
Vaccines	Vaccines to prevent disease in humans	[88]
	Novel approaches to using vaccines to prevent infection in humans and other animals	[100, 106, 132]
	Vaccines for cats to prevent oocyst shedding	[96, 97]
Environmental Approaches	Novel approaches to prevention of contamination of the environment by oocysts	[49]
	Water purification	[63]

### Clinical Manifestations of Ocular and Congenitally Infected Children and Treatment with Anti-parasitic Medicines

#### Panama

**Fig. 1:**
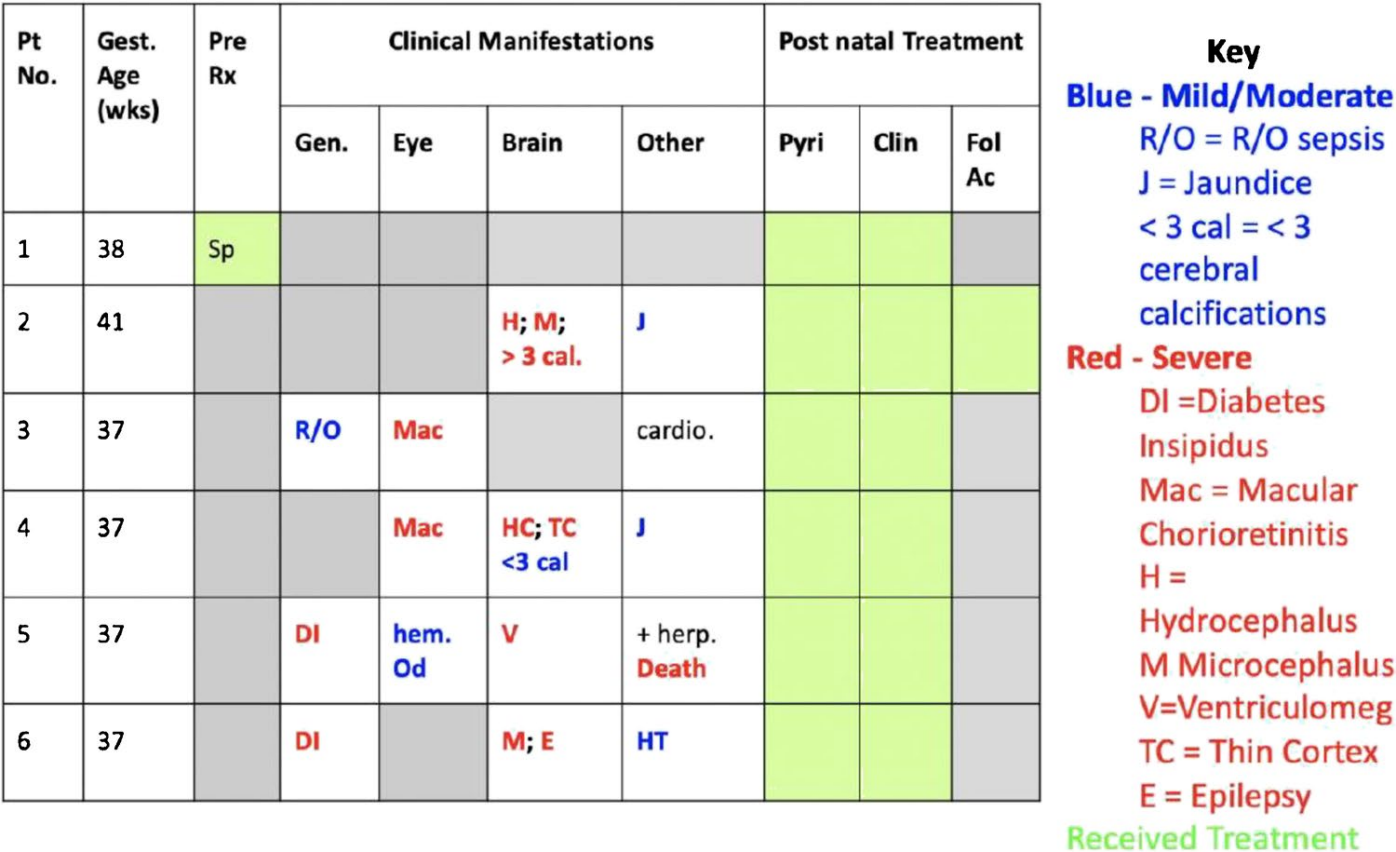
Results of characterization of the six congenitally infected children in Ciudad de Panama who presented for care at the new perinatal infections program in 2016-2017. The only asymptomatic child of the six (patient 1) corresponded to the only mother who was screened during gestation and given prenatal treatment with spiramycin. The other, unscreened mothers gave birth to children who had clinical manifestations such as hydrocephalus and diabetes insipidus

Details of the education and epidemiology programs were provided in Parts I to III of this series. They are a critical part of building this overall program. In this context, a physician (XN) and a visiting student (MR), initiated a retrospective chart review of patients who were identified through recognition of clinical symptoms and signs or were identified in a serologic screening program. The infants had a diagnosis of congenital toxoplasmosis made between 2016-2017 and were known to the Pediatric Infectious Diseases experts at Hospital del Niño, Panama City, Panamá. This work was performed with permission from the hospital’s bioethics committee. For each of six congenital toxoplasmosis cases identified, data regarding prenatal and postnatal treatment, gestational age at birth, clinical manifestations and laboratory tests were collected (Fig. 1; also See Ramirez et al. in the Supplement referring to serologic screening in Panamá, a glass half empty and a glass half full). These cases also were included in and formed the basis for a later, more extensive review [59•]. Furthermore, a perinatal healthcare center was established at Hospital del Niño and a program with skill in retinal disease and uveitis was developed. A gestational screening program to identify seroconverting pregnant women, facilitate initiation of treatment and prevent transmission to the fetus was established with an IRB protocol awaiting approval to provide the foundation for a study, which can be initiated when the complications associated with the SARS-CoVi-2 pandemic permit. This disease was recognized as a significant public health problem in Panamá’ and has become a focus for health care, public health and scientific initiatives in the country, extending the present studies described herein.

#### Colombia

##### Overview

In the spatial epidemiology analyses as described in Part I, additional ongoing studies were included [62••, 63, 64.••••, 65]. Guidelines for treatment nationwide were adopted and markedly reduced severe disease and prevalence of the infection were noted in a study extending from 2001 to 2019 [66••]. A new program to evaluate point of care testing in Armenia, Colombia was implemented [Gomez -Marin, In Progress 2022].

##### Pregnant Women and Congenital Toxoplasmosis

These studies include evaluations and treatment of seroconversion of seronegative pregnant women and their infants. A study of a point-of-care (POC) test developed in France, the United States and Morocco [43••, 44••, 57•, 58••] was also initiated. This is now being used in Europe with CE mark (European FDA equivalent) approval.

##### Ocular Toxoplasmosis in Adults

In addition, patients with known ocular toxoplasmosis have been identified and active infection treated. A free evaluation was offered in several areas in Quindío, Colombia, and patients who were seropositive with typical ocular lesions of toxoplasmosis were identified and referred for further care to a Center in Quindio, Armenia. This work was described in a recent separate paper [63].

#### United States, Europe and Morocco

##### Introduction of ICT-LDBIO Point of Care Test in Europe, the United States, Morocco, Colombia and Panamá’

In France, the Point of Care test was used primarily for sera but is now CE Marked. In the United States an ongoing longitudinal study called the National Collaborative Chicago-based Congenital Toxoplasmosis Study (NCCCTS) was initiated in 1981 and continues into the present time was reviewed in Part I (1-150). This study has been central to characterizing and developing treatment for this disease. In Part IV we address newer work with Point of Care testing to make the diagnosis earlier so treatment of the pregnant woman can prevent infection or treat the fetus.

The LDBIO POC test was developed and first tested in France, then the United States, Morocco and then Panamá. Testing in Colombia has been initiated in the past month. This process and findings are described herein.

The test utilizes 15 microliters of sera or 30 microliters of whole blood placed in a well overlying filter paper. Four drops of elution buffer are added. Along the filter paper, there are black beads coated with *Toxoplasma* antigen that makes a double sandwich to capture human anti-*Toxoplasma* serum antibody and creates a black line when antibodies are captured. There is also a blue bead that is coated with goat anti-rabbit IgG that reacts with a line of rabbit IgG and serves as a migration control. This test detects both IgG and IgM antibodies specific for *Toxoplasma*.

The LDBIO lateral immunochromatography test was performed using sera as described in Begeman et al, and with whole blood from a finger prick by Lykins et al [43••, 44••]. In the first part of this analysis in Panamá’, one hundred sera were randomly selected from a larger number that were tested with the Roche IgG and IgM tests in Panamá’, as described in Part III of this series of manuscripts. These were randomly selected serum samples from the group of women who had serologic screening as described in Part III of this series of manuscripts at Hospital Santo Tomás, Panamá’ City, Panamá’. The investigators in this study randomly selected sera collected during this study that were sent to the United States’ Chicago center where the LDBIO test was performed by a visiting scholar (AG). These samples had been sent to the Toxoplasmosis Center at the University of Chicago and the Toxoplasmosis Research Institute where they were tested with the LDBIO *Toxoplasma* ICT IgG-IgM. A subset of the sample that were discrepant in the Roche and Chicago testing were also tested in Lyon France with the LDBIO Toxo II WB. LDBIO Toxo II WB, considered as a gold standard in Europe, was performed as per manufacturer’s instruction. The lateral immunochromatography test LDBio Toxoplasma IgG-IgM demonstrates nearly 100% specificity and sensitivity when tested compared with the Sabin - Feldman dye test or other comparable tests.

To introduce the use of this LDBIO chromatographic test that meets WHO-ASSURED (affordable, sensitive, specific, user-friendly. rapid and robust, equipment-free, deliverable), to Panamá’, the test was first introduced in an established study on wellness and aging in Panamá’ City, a high-priority program in which sera were obtained. The Panama Ethics Committee reviewed this study and gave permission to test the bio bank of sera using the LDBIO test. This second set of tests with sera in Panama was performed at the translational center at the Panama ‘ National Institutes of Health (INDICASAT). Sera were obtained from participants in the Panama Aging Research Institute (PARI) 1 and PARI 2 wellness and aging studies and had been stored at -80 degrees Fahrenheit. Participants were 65 years of age or older. A separate report will describe additional cognitive testing which was performed in that study (Britton, McLeod et al, In preparation in 2022.) Herein we only address prevalence of antibody using this now CE Marked test.

In the United States, France and Morocco, more than three thousand sera/persons have been tested using the LDBIO *Toxoplasma* ICT IgG-IgM test kit, with over one thousand of these also performed with whole blood from fingerstick testing, as described by Lykins et al [44••]. The results from Morocco have been published separately in an epidemiologic study that compared results with the LDBIO-POC test and the Pasteur Institute BIORAD ELISA [57•].

##### Anti-parasitic Treatment

As mentioned in Part I, pyrimethamine, sulfadiazine, leucovorin and spiramycin are essential for the treatment and prevention of congenital toxoplasmosis [17••, 33••, 46•, 48••, 53••, 60••, 65]. Through ongoing programs for care, the personal experience of the authors informed observations of availability of medicines in Panamá’ and the United States. These observations were made in the context of building programs in each of these countries.

##### Marginalized Populations

A challenge for delivery of care in all three countries—Panamá’, Colombia and the United States—involved considering how to reach out to, test and treat patients in marginalized populations. Recently, it is also clear that the same difficulties also exist in Canada, for example for the Inuit people in the Arctic. This has been especially problematic and evident during the COVID-19 pandemic in the United States and its territories. Poverty has made access to healthcare including medicines more difficult and has stressed the healthcare system, augmenting problems associated with access, availability of medicines and care that were substantial before.

In a complementary analysis, four students (two medical students and two social policy students) performed an analysis of how to develop suitable strategies for care of the Embera indigenous population in Panamá’. The Embera live in close proximity to soil, wild cats, potentially contaminated water, with an average lifespan of approximately thirty years, making substantial risks for acquisition of *Toxoplasma*. Some possible approaches considered included optimizing the roles of the public health system, MINSA, regular delivery of healthcare to remote areas by medical teams, developing systems to provide medicines and help patients reach areas with more sophisticated care, and systems to monitor treatment in such populations (Part IV. Supplemental Part A). This is also a consideration in indigenous populations in the United States and Canada, the problem including frequent infections for those in remote areas of the Arctic region.

## Special Considerations

### Clinical Manifestations of Congenitally Infected Children and Treatment with Medicines

#### Panama

For Panamá’, summaries from an initial study of the six children diagnosed with congenital toxoplasmosis at Hospital del Niño in 2016-2017, showed that five were severely affected (see Fig. 1). The only asymptomatic child corresponded to the only mother who was screened during gestation and given prenatal treatment with spiramycin. The other five mothers, who were not screened, did not receive any prenatal treatment, resulting in both severe clinical manifestations—such as hydrocephalus, microcephaly, diabetes insipidus, chorioretinitis involving the macula—and mild to moderate clinical manifestations, such as jaundice and cerebral calcifications. This is not an association between prenatal treatment and subclinical disease. Although prenatal treatment prevents sequelae, in this case, the way these patients were identified represents two completely different populations. Those symptomatic children were only identified because they had symptoms, while the asymptomatic infant was identified by prenatal screening. These data do not elucidate how many asymptomatic patients were born in the same period of time but had not been treated during prenatal care. Prenatal care would have benefited those babies with neurological disabilities. Also, many babies may have been born asymptomatic, to mothers who were contaminated by T*oxoplasma* in the last months of gestation, but were deprived of prenatal and postnatal treatment, which could have avoided sequelae, mainly ocular, that will only be noticed later in their life.

**Fig. 2:**
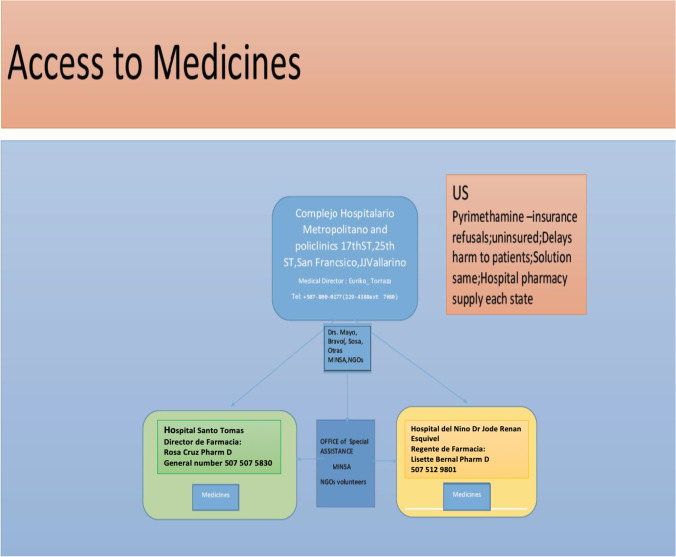
Model of a distribution scheme to multiple hospitals with a central distribution site for medicines that can be used to treat toxoplasmosis. As distribution of medicines can be a significant issue for any country, this figure also addresses challenges with supplying medicines such as pyrimethamine in the United States

A medicine distribution strategy was created but is not yet implemented fully. This was because one area of difficulty noted when these early patients were identified in Panamá’ was obtaining medicines due to limitations with importation (Fig. 2).

**Fig. 3:**
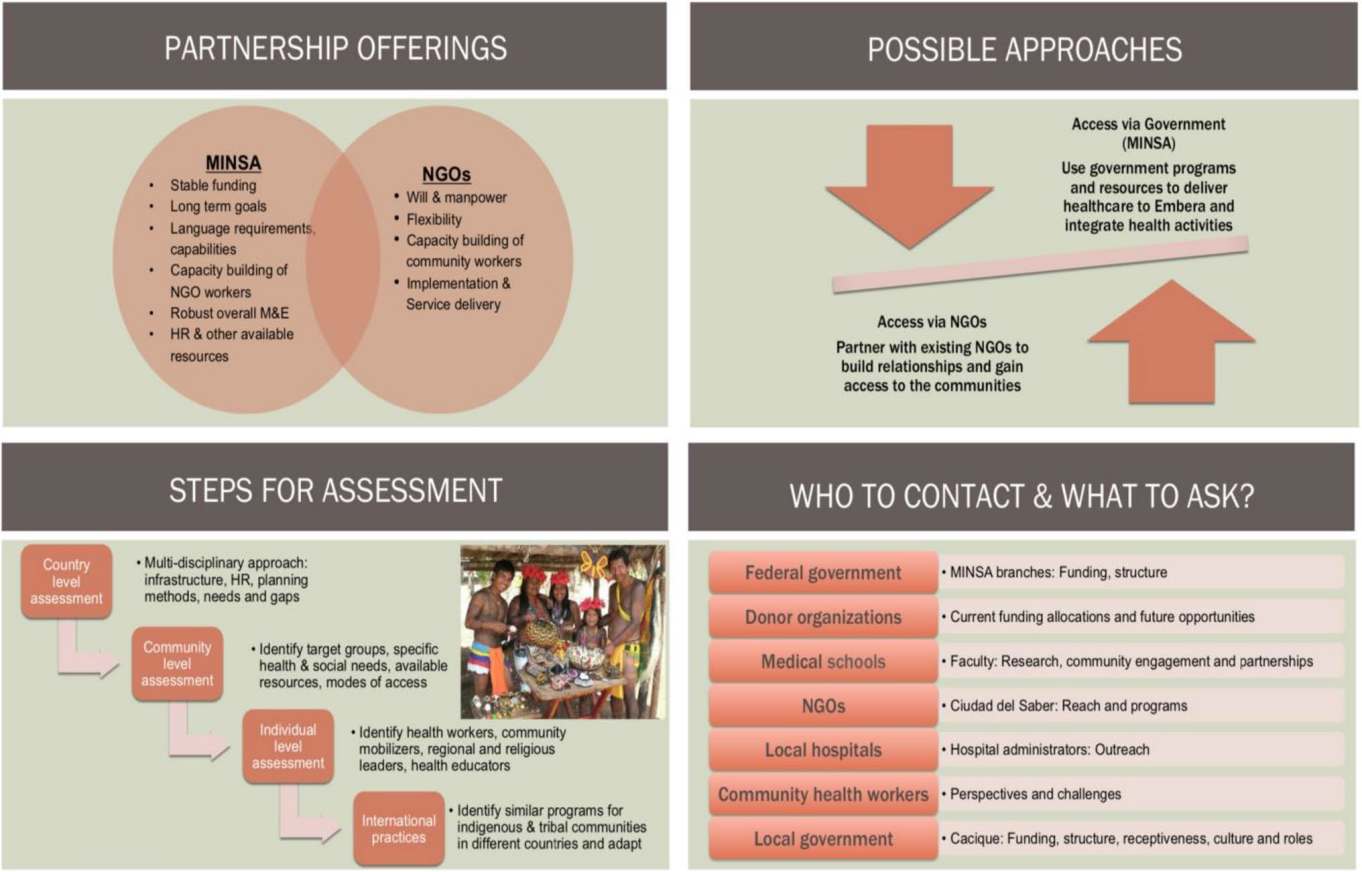
Vishan Dhamsania, Nick Graves, Marci Kirchberg and Kopal Mathur considered international development strategies for bringing toxoplasmosis screening and treatment to the Embera, an indigenous group in Panamá. The student group evaluated what strengths and weaknesses of the Panamanian Ministry of Health (MINSA) and non-governmental organizations (NGOs) would be to implement screening programs for toxoplasmosis for the Embera. They considered the following criteria for successful implementation for a program: politics, economics, culture and sustainability. They found complementary strengths from MINSA and NGOs

Considerations of ways in which government programs and NGOs could work to help indigenous and marginalized patients were considered by a group of medical students and public policy graduate students. The strengths of both the government agencies and NGOs were noted to be complementary (Fig. 3, Part IV Supplemental Part A).

#### Colombia

In an earlier manuscript [48••] the dramatic effect on reducing illness and disease in Colombia by introducing screening has demonstrated efficacy and utility of public health programs [48••]. Nonetheless, women in the health care system delivering infants at one hospital had little knowledge but easily learned relevant materials with educational materials as presented in Part II of this series of manuscripts.

Concerning bottled water and uncooked meat exposure, a reciprocal association was noted. In Guaduales de la Villa, the habits of drinking bottled water and eating undercooked meat were negatively associated with infection. At first this seemed counter intuitive and contrary to earlier studies. To better understand these results, a closer look at methodology, in the context of the results, present a possible set of explanations demonstrating possible relative risks and efficacy of public health measures: Eating undercooked meat was coded as 1 for 'yes' and O for 'no' . Presence of *Toxoplasma* specific IgG was coded as 1 and its absence was coded O.

This analysis used a logistic regression in R with seropositivity as the outcome and ***all*** variables used as covariates. In this model, undercooked meat had a *p* value of 0.034. In an analysis where undercooked meat is the *only* predictor and other variables are not accounted for, however, it was not significant with a p value of 0.06. The total model better adjusted for the effects of the other variables and the analyses in context of each other suggest that oocyst contamination of water is the most important source of infection, with both variables bottled water and less well cooked meat consumption perhaps influenced by socioeconomic status [57•]. Another separate statistical analysis of the same data set confirmed the latter finding. This analysis consisted of a simple analysis of the differences in proportions by using the chi-squared test or Fisher's exact test, as appropriate. Odds ratios and 95% confidence intervals were calculated. SPSS version 25 (SPSS Inc. IBM, Chicago, USA) statistical program was used to perform the analysis. P values <0.05 were considered statistically significant. As a single variable, the p value for undercooked meat was 0.057, similar to the analysis herein using R.

A previous study in Armenia concerning transmission by meat, suggests that it could be possible that people who prefer rare meat buy at stores where the meat is frozen [97]: A scoring system that ranked hygienic conditions and freezing of meat was utilized to compare meat stores. A score of 1 was least and a score of 3 was most. In stores that were most hygienic and froze meat, PCR for *T. gondii* DNA was least in meat samples. For chicken in the most hygienic stores, PCR detecting *T. gondii* DNA was significantly less than in the less hygienic stores. Another possible explanation could relate to socioeconomic status that allowed affording the more hygienic, less contaminated source of meat and also be a surrogate marker for another variable like adequate funds for bottled water.

#### United States

##### NCCCTS and Treatment

**Fig. 4.**
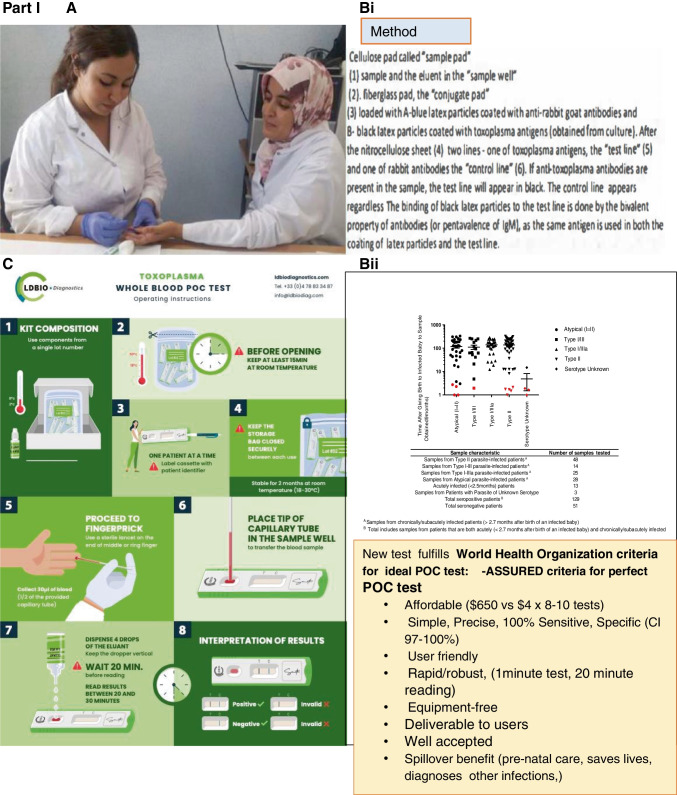

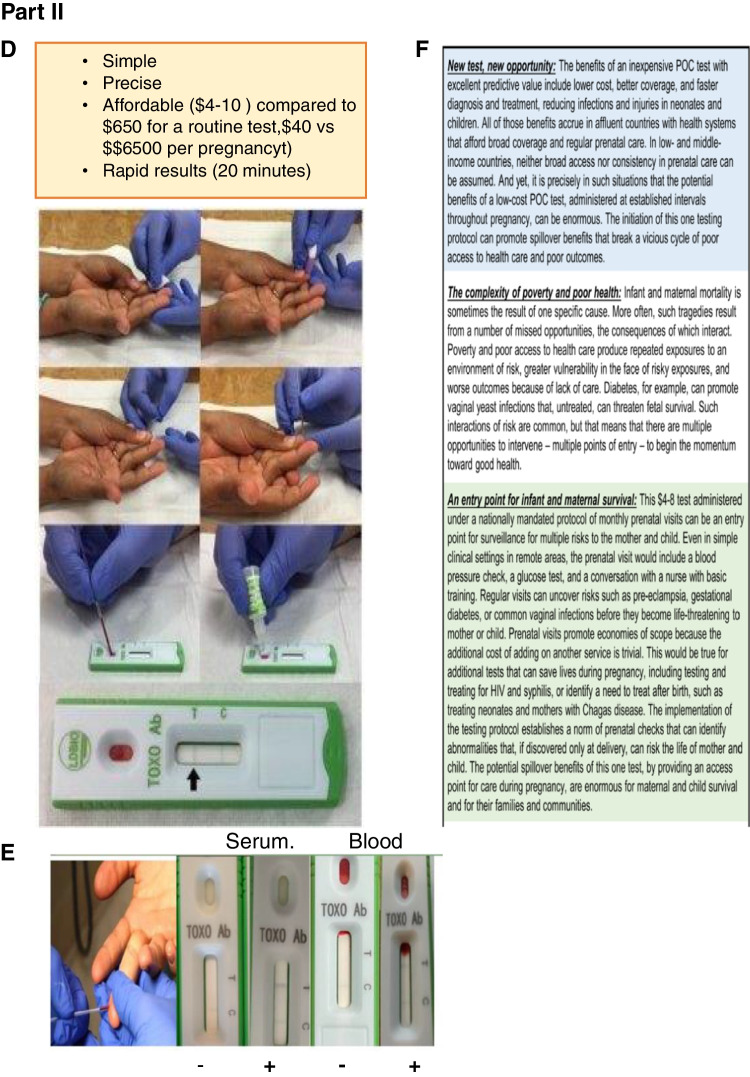
Testing of the Lateral Immunochromatography G and M test with whole blood from finger stick. **Part I:** A. Method used in study. B. Description of early results (from PLoS Neglected Tropical Diseases with permission). C. Brochure that will be the instructional material for when this is used in clinical practice, as in package insert shown with instructions for use of kit. Images show method used step by step. **Part II:** D. Representative results for serum or whole blood in this test. E. Example of obtaining blood by fingerstick, example of positive bands(T) and negative result (no T but C-control-band, C, sera , left, whole blood -right. F. Our expectation is that this global initiative to address the significant health problems toxoplasmosis presents, as a focus of the programs being built, will have spillover benefit to promote well-being, improve water supply reducing other water borne diseases, in addition to preventing devastating consequences of this infection. The opportunity for spillover benefit for diagnosing and treating other diseases, and the opportunity for understanding basic, translational aspects of the biology, developing vaccines and better medicines are another substantial benefit of this global initiative. Bii, D, F. With permission PloS Neglected Tropical Diseases, E. UChicago, article by  John Easton

Results from work in the United States were reviewed in Part 1 of this series of papers [1••, 2, 3••, 4••, 5••, 6••, 7••, 8••, 9••, 10••, 11••, 12, 13, 14••, 15••, 16••, 17••, 18•, 19•, 20•, 21•, 22•, 23, 24••, 25, 26, 27•, 28•, 29–30, 31•, 32•, 33••, 34•, 35•, 36, 37•, 38•, 39••, 40•, 41•, 42••, 43••, 44••, 45, 46•, 47••, 48••, 49, 50•, 52, 55••, 56••] but not with an overview in some time. Thus this data set is included herein in a not previously published composite form in a table with comments that summarize some of the major findings in the context of others work (Part 1: Table 1; Fig. 4, 1-51). The cost benefit analyses emphasize that treatment brings not only reduction in individual suffering but also brings substantial cost savings for countries (e.g., 14-fold in Austria). The major role France has had in developing this improved approach that saves life, sight and cognition is represented also in Part 1: Fig. 4. As this has been addressed in earlier publications also included in the references, it is not considered in more depth here.

### Updates

#### Screening Programs in the United States

Most diagnosis in the United States is made when there is symptomatic illness and obvious clinical manifestations and even then diagnosis is often missed or delayed. Screening during gestation occurs in small numbers of “high end” obstetrical practices but occurs only rarely on a larger scale or systematically in the United States. In contrast, this is usual now in France, Austria, Brazil, Colombia, Morocco among other countries.

Massachusetts and New Hampshire perform newborn screening [76]. Screening by obstetricians is infrequent and not systematic, with some higher end practices screening.

The early studies of the NCCCTS in the U.S. also confirm the findings in France [33••], that , although postnatal treatment confers considerable benefit, prompt prenatal treatment of seroconverting women was associated with the best outcomes and obviate the effect of parasite strain [33••].

Cost benefit analyses creating mathematical models that now have been applied in other countries were performed [56••]. Work with Point of Care (POC) testing grew from a parent motivated initiative. This occurred as follows: parents who understood the success of the screening initiatives in France and Austria aimed to prevent congenital toxoplasmosis through instituting a similar program in the US [51•, 52, 53••, 60••]. They sought to incentivize the legislature in Illinois to pass a law similar to the laws that were passed in France and Austria mandating serologic screening programs for acquisition of *Toxoplasma* by pregnant women. The coordinator at the Toxoplasmosis Center, M Sautter, worked with a legislator in Springfield, Illinois, to write a law that said obstetricians should inform their patients about *Toxoplasma* and offer testing [61]. A group of patients, their families and physicians involved with the NCCCTS traveled to Springfield, Illinois and met with the special Committee on Healthcare (Part IV Supplemental Part B). The legislators wanted to pass the law, while the Department of Public Health emphasized the importance of having an infrastructure in place as a public health program, while both the Obstetrical Society and the Department of Health sought a cost-benefit analysis to establish feasibility and assess possible costs versus benefits from such a program in the United States.

Some members of an organization concerned about terminations of pregnancies initially wanted to block this legislation but quickly realized that this was a program to diagnose and treat to prevent the infection and its serious sequelae rather than causing any loss of life. They became strong supporters of the legislation during and after the hearing. The Obstetrical Society felt that education programs rather than legislating what doctors should do was a better approach.

Prompted by this experience, cost-benefit analysis algorithms were developed [56••]. They have been applied in the United States, Austria and France as discussed in more detail below in the following section [51•, 52, 53••, 56••, 60••]. Educational materials were developed as presented herein in Part II of this series, as well as earlier in text books, scientific and clinical teaching presentations, and in many different publications, blogs, websites and public service announcements throughout the past 40 years. These are being made freely available on a not-for-profit website (Toxoplasmosis.org), in a University of California at RadioBio.net and now NSF sponsored podcast :(https://ucmerced.box.com/s/gtnu1gujkipfyc9fewmqi7rqy1nekjr2); (NSFScience360). This work has recently taken into consideration a new Randomized Control Trial in France by Mandelbrot et al showing benefit of pyrimethamine, sulfadiazine, leucovorin treatment after 14 weeks of gestation beginning dating gestation with onset of amenorrhea [60••]. The possible benefits of screening during pregnancy in the United States and practical aspects of its implementation are being adapted and considered in the United States at the Toxoplasmosis Center in Chicago and with support by the Thrasher Children’s Charity and the Kiphart Global Health Foundation [61]. Similar studies are taking place in Morocco and Colombia and planned for Panamá’ [57•, 59•] with interest in other countries. This POC test is now approved in Europe with CEMark (FDA equivalent for Europe) based on analyses in the U.S. France and Morocco (Tables 1, 2, 3, and 4). This work has been/will be submitted for consideration by the FDA for 510K and dual CLIA waiver in the United States (Summary in Table 4). This review will take place as soon as the extensive approval process for emergency use applications for SARS-CoV-2 no longer causes deferral of other types of FDA reviews in the United States.

#### Introduction of LDBIO IgG/IgM Lateral Chromatography Point of Care Test

Details of the use of this test have been described for France, the United States and Morocco. Using serum and whole blood, we have found high functioning of this lateral chromatography test with essentially 100% sensitivity and specificity, meeting the WHO-ASSURED criteria, with over 3,000 people now tested. [43••, 44••] (Tables 1, 2, 3, and 4).

Recent presentations were at International meetings, in teaching sessions, on a University of California (Merced) podcast, on a National Science Foundation (NSF) podcast, and as a plenary session keynote student presentation.

Links to International ToxoXV meeting in January 2021 are at toxoplasmosis.org (Dr. McLeod's Presentation: https://vimeo.com/showcase/8031816/video/502588615); (Dr. EL Bissati's Presentation: https://vimeo.com/showcase/8031816/video/502601087) and to the podcast discussed above at .https://ucmerced.box.com/s/gtnu1gujkipfyc9fewmqi7rqy1nekjr2 which include descriptions of this work.

Instructions are located in the kit, in publications, and are available at toxoplasmosis.org along with other educational materials herein (Fig. 4 and pamphlets).

Testing of more than 3000 serum samples in the United States, France and Morocco provided the results shown in Fig. 4 and Table 4. The LDBIO device demonstrates nearly 100% specificity and sensitivity when tested against gold standard Sabin-Feldman dye test or other comparable tests used with sera in these three geographic settings. This lateral chromatography test has become CE marked in Europe. Similarly, over 1000 sera were tested with whole blood obtained with a fingerstick in the United States and Morocco. Specificity and sensitivity demonstrated exceptionally high performance, as described above, for this test. In the United States, use of the POC test monthly beginning before conception, or early in the first trimester and continuing to the 6^th^ week postpartum visit was extremely well received by patients, obstetricians and nurses, as demonstrated by questionnaires (Lykins, Leahy, Zhou, Siddiqui, Leong, Goodall, Romero, Ismael, Christmas, Peyron, Wallon, McLeod, in preparation, 2022). These data and others supported the CE Mark approval in Europe (parallel to the FDA in Europe), and data are waiting for review for 510(k) clearance and dual CLIA waiver with the FDA and CLIA in the United States. The FDA has put all reviews of diagnostic applications on hold to prioritize emergency use authorization application for COVID-19 diagnostics, vaccines and treatments. This material will be reviewed when clearance procedures can resume without the need to prioritize testing for reagents and methodology that will improve outcomes for SARS-CoV-2. This is anticipated to occur in 2022. The next step is to make certain that this high performing test can be utilized as reliably by less experienced practitioners. This is being prioritized currently in different demographics and at scale.

This test does solve the cost issues (between $5-10 per test meeting all WHO ASSURED criteria), specifically, and solving problems with false positive IgM results from commercial tests) (Grose, Wallon, Chapey, Leahy, Zhou, Piarroux, Limonne, Houze, Abraham, McLeod, In submission, 2022).

In working with the LDBIO *Toxoplasma* ICT IgG-IgM (LDBIO POC) in Lyon it was found that for 11 sera from patients with malaria there were no false positives. From 51 patients with leishmaniasis there were 6 false positives. These analyses were performed separately from those mentioned above in the United States, France and Morocco and were specifically sera from patients who were suffering from leishmaniasis in countries that are endemic for leishmania infections. As indicated in the Instructions for Use of the kit this could be a confounding factor for serodiagnosis for *T.gondii* with the LDBio for patients with leishmaniasis.

In Panamá, 100 sera from pregnant women were tested with the Roche test; 50% were positive. Testing of a small subset of these samples with the LDBIO test when the sera was shipped from Panama revealed three false positives of these samples when compared with Western blot, different than the prospective testing performed in the United States, France and Morocco where there was nearly 100% sensitivity and specificity. This latter result possibly was due to handling of the sera before it arrived in the United States or with a separate population which might result in separate concomitant diseases in Panama relative to the United States.

In an aging population of individuals over 65 years of age in Panama City, seroprevalence was found using the LDBIO test to be 85% with this CE Marked now approved test. Studies are underway to determine if *T. gondii,* this billion-brain parasite, contributes to neurodegeneration or other chronic diseases that are increased in aging populations (Britton, Villareyes, Perez, Naranjo-Galvis, Wroblewski, Karrison, Dogra, Ramirez, Dovgin, Dovgin, Fraczek, Lorenzi, Bennett, Wang, Kim, Funk, Zhou, Dodya, Ross, Piarroux, Limonne, McLeod, et al 2022, manuscript in preparation).

In Panamá, ethics committee materials are prepared and a prospective study of 200 pregnant women with monthly screening using the LDBio test is planned (Charter, Reyes, Ashti, Grose, McLeod, et al 2022, in progress), awaiting a diminution of cases of SARS-CoV-2 in Panamá’. This study was deferred because of high numbers of SARS-CoV-2 cases in Panamá’.

A similar study is being initiated in Colombia currently. Pregnant women are being tested monthly in a screening program to identify those who seroconvert and thus are at risk of transmitting the infection to their fetus. Identification of seroconverting women will allow prompt initiation of treatment to prevent the infection. This approach has already been found to reduce morbidity and mortality from this infection using standard commercial tests. The LDBIO POC, if sensitive and specific in this setting, can substantially reduce the cost of this type of program.

There are additional similar data from Morocco. In Morocco, a similar study was performed, and the findings are presented in Tables 1 and 4 [57•]. Specifically, in diverse settings, a total of 632 women were studied. Initially, for 283 women, sera were tested by Platellia ELISA IgG and IgM along with LDBIO POC fingerstick whole blood test. Then, for 349 women, a study was performed that compared POC testing with whole blood obtained by fingerstick and serum from contemporaneous venipuncture. Comparison of the results of the same sera tested with western blot also was performed. POC test sensitivity was 96.4% [IC95 90.6-98.9%] and specificity was 99.6% [IC95 97.3-99.9%]. Prevalence of *Toxoplasma* infection among women living in rural and mountainous areas, and in urban areas with lower educational levels, was high. Exposure to soil, agricultural work, well water and not washing fruits and vegetables prior to consuming them were risk factors for the 632 women within all the settings [57•]. Thus, in Morocco, sensitivity and specificity of the lateral chromatography test were again exceptional [57•].

In Armenia, Colombia, a prospective study to screen 200 pregnant women has been initiated with support and reagents approved for entry into the country (Gomez-Marin et al, unpublished 2022).

#### Anti-parasitic Treatments using Medicines Currently Available in Clinical Practice

In all three countries currently, accessing medicines presents challenges. The recent history concerning the availability of medicines has influenced care for toxoplasmosis in the United States, as discussed in Part I. This most recently has involved pyrimethamine and sulfadiazine.

In Panamá’, access to medicine is a challenge due to unavailability of medications. Pyrimethamine remains one of many essential medicines where importation is complicated. A hypothetical system was designed and created (Fig. 2) which centralizes and makes low-cost medicines available 24/7. It is hoped that along with creation of Centers with expertise in care, access to medicines will improve. There are some areas in Panamá, in Chiriquí for example (see Pirla et al in Supplemental in Building: Spatial Epidemiology Part III) or regionally within Panama City like San Miguelito where there is a particularly compelling need for availability of care due to the higher prevalence of contact with soil, poverty and dirt floors in dwellings.

In Colombia, similar difficulties with access are evident for certain marginalized populations. This is also confounded by oocyst contamination of water sources. The availability of bottled water is likely correlated with socioeconomic class.

#### Marginalized Populations

In Panamá,’ medical care for indigenous populations presents a particular challenge. Two medical students and two public policy students from the University of Chicago prepared a capstone course project (supplemental figure). One of the medical students worked for a year with the Ministry of Health (MINSA) in Panama on healthcare initiatives for the Embera people. Another of the students had worked to provide medical care in the region of Nigeria where Boca Haram was active while in the Military. Other students had worked with the Peace Corps, so each brought unique perspectives. Discussions included the Director of the Initiatives for Toxoplasmosis in Panama with legal background. Other discussants were the Director and Nurse in the Whiteriver Indian Health Service Programs in Arizona. The students performed an analysis of the strengths and weaknesses of public health policies and care algorithms when public agencies and not-for-profit agencies participated in addressing care for marginalized populations (Fig. 3; Supplemental A for Part IV). They concluded that each approach brought strengths, some overlapping. Some strengths from the governmental programs were: ability to uniformly assist with care in a broad, well-organized manner with less limitation of resources. Strengths for the NGOs included ability to assist families directly, flexibly and efficiently. These two differing approaches complement each other. The combination of both methods of supporting care could help to optimally deliver such care to marginalized populations who live in remote areas with more limited access to care such as to the Embera peoples in Panamá’. This analysis provided a roadmap for organizing such care (Fig. 2). A challenge arises from introducing a screening program without clear, immediate benefit for a population that has an average lifespan of 30 years. This is especially the case if they are fatalistic about illness in infants and what life can hold for a severely ill infant. This can make motivating screening programs seem irrelevant when there is no profound, clear, immediate evidence of benefit.

In the United States, although this is an infection affecting persons across all demographics, some of the populations of concern include people living in poverty [73••], individuals experiencing homelessness and some rural/agrarian populations.

There is considerable recent lay press concerning the poor record for maternal and child health and well-being, health care disparities, including for the sentinel clinical findings of toxoplasmosis, e.g., , prematurity, small for gestational age, intrauterine growth retardation, death in the first year of life, maternal morbidity and for marginalized indigenous and Latino populations. Titles from recent (November, 2021) lay press news articles emphasize this problem in the U.S.. These titles include: “U.S. Maternal And Infant Mortality: More Signs Of Public Health Neglect” (Forbes**);** “The U.S. Remains One of the Most Dangerous High-Income Countries for Childbirth, According to a New Report” (Health); Opinion (Apple News) | The lives and deaths of infants: America's disturbing disadvantage”.

Another example that prevalence varies by demographics is that seroprevalence in the Lancaster Amish area among those of childbearing age is approximately 50% with high risk and high exposure for those who are seronegative [32•] in contrast to other areas in the US where prevalence is <15%. Influence of other aspects of demographics is evident in the US in NCCCTS studies (e.g., 33) and in our large data analysis (45). Geography, ethnicity and socioeconomic status were associated with parasite serotype as was anatomy of hydrocephalous, prematurity and severity of illness at birth. Treatment *in utero* appeared to obviate those associations (33).

#### Need for Novel Medicines that Eliminate Encysted Bradyzoites and “Persister” Organisms, and Vaccines that Prevent this Infection in Humans and Oocyst Shedding by Cats

**Fig. 5:**
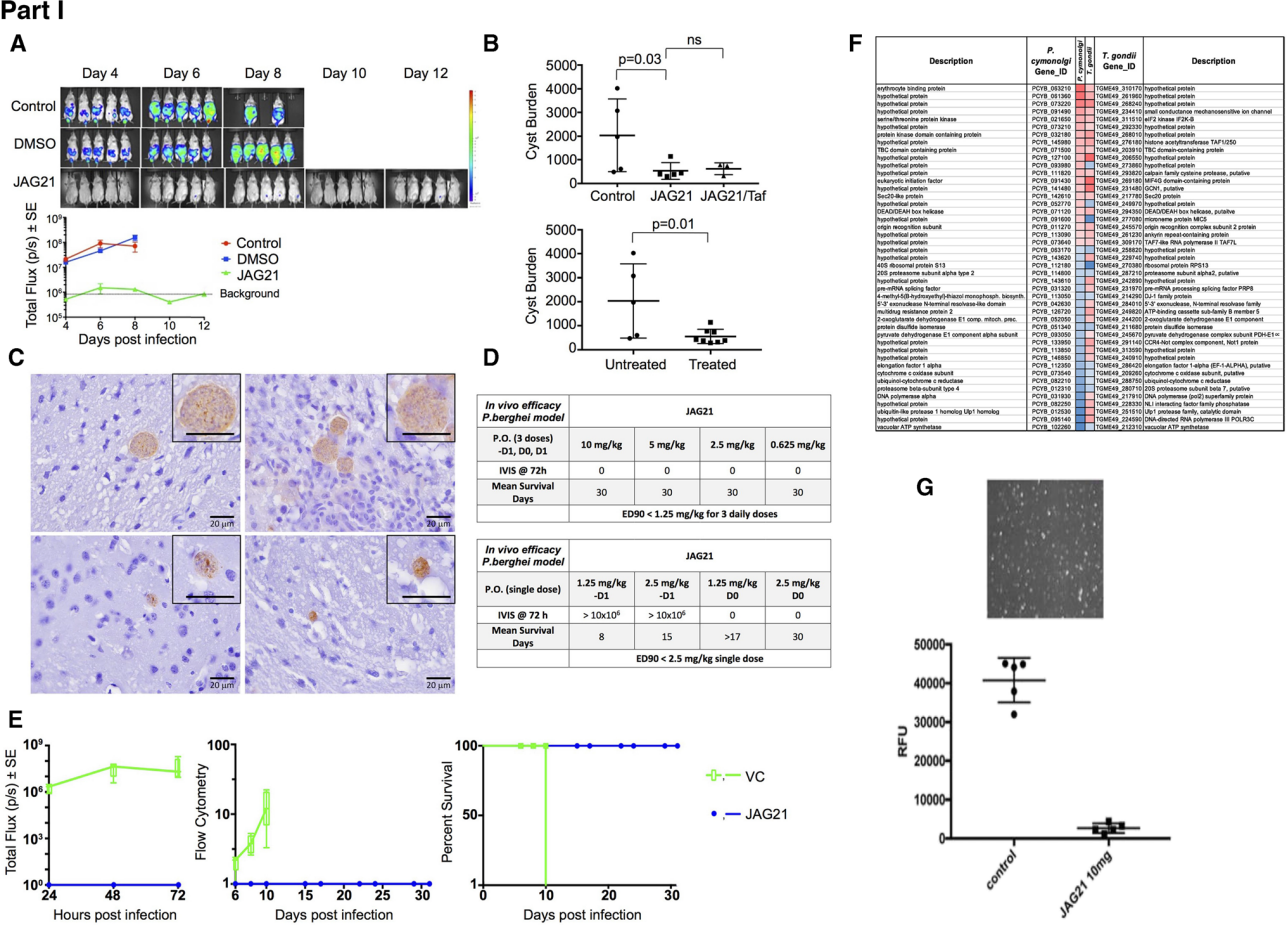

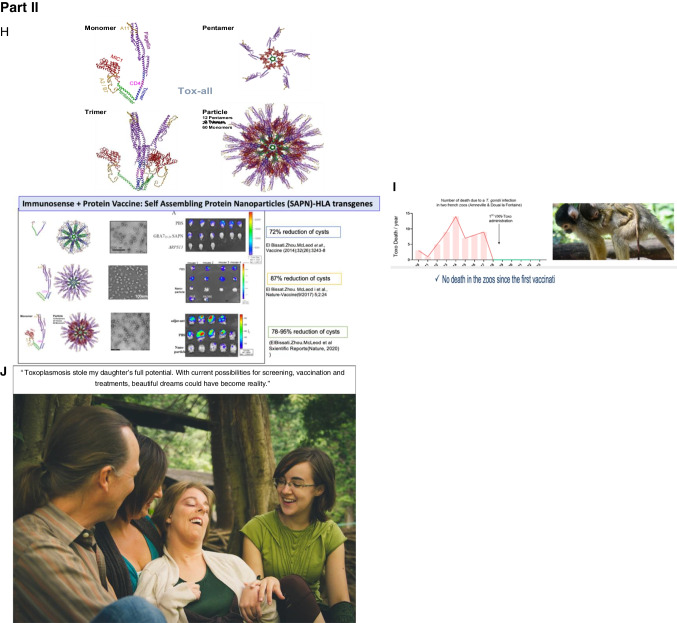
The Future. Recent studies in Chicago to develop medicines and vaccines, and an intranasal nano-vaccine and a dendrimer RNA vaccine developed by others (I). Part I. A potent tetrahydroquinolone effective against tachyzoites and bradyzoites *in vitro*, synergistic with atovaquone, eliminates >95% of encysted bradyzoites in vivo,, while active with a single oral dose of a nano formulation against tachyzoite infection and *Plasmodium berghei’s* three life cycle stages. Specifically reproduced with permission from Frontiers Cell Infection Microbiol : Figure and Legend directly from this paper. “JAG21 is a mature lead that protects against *Toxoplasma gondii* and *Plasmodium berghei in vivo*. **(A)** JAG21 treatment for 14 days protects against *T. gondii* tachyzoites *in vivo*. Tachyzoite challenge with Prugneaud luciferase parasites imaged with leuciferin using IVIS demonstrates that treatment with JAG21 eliminates leuciferase expressing parasites and leads to 100% survival of JAG21 treated infected mice. No cysts were found in brains of mice at 30 days after infection when they have been treated with JAG21 for the first 14 days after infection. There were 2 biological replicate experiments with 5 mice per group with similar results. **(B)** JAG21 and JAG21 plus tafenoquine markedly reduce Me49 strain brain cyst numbers *in vivo* in Balb/C mice at 30 days after infection. Parasites were quantitated by scanning the entire immunoperoxidase stained slide in an automated manner and by two observers blinded to the experimental treatment using microscopic evaluation. In each of two experiments, the numbers of mice per group were as follows: Experiment 1 had 4 diluent controls, 5 JAG21, 4 JAG21/Tafenoquine treated mice; and Experiment 2 had 5 diluent controls, 5 JAG21, 3 JAG21/Tafenoquine treated mice. Immunoperoxidase staining was performed. Parasite burden was quantitated using a positive pixel count algorithm of Aperio ImageScope software. Positive pixels were normalized to tissue area (mm^2^). Quantification was by counting positive pixels per square area. The entire brain in one section was scanned for each mouse. The parasite burden was quantitated as units of positive pixels per mm^2^. The average ± S.E.M. numbers of mm^2^ per slide quantitated was 30.2±1.6 mm^2^ per mouse for this quantification. Each high power field of view shown in **C** is ~0.02 mm^2^ per field of view. A representative single experiment is presented and the data from the two experiments analyzed together also demonstrated significant differences between the untreated and treated groups (*p* < 0.01; Supplemental Figure 1). **(C)** Microscopic evaluation of the slides reveal effect of JAG21 and JAG21 plus tafenoquine having the same pattern as the automated quantitation of immunoperoxidase stained material. There are usual appearing cysts in the DMSO control untreated mice as shown in the top panels, and rare cysts in the treated mice with most of the brown material appearing amorphous (bottom panels). **(D)** JAG21 nanoformulation dosages administered to *P. berghei* infected C57Bl6/albino mice compared with vehicle control. Design of single dose and 3 day dose experiments. **(E)** JAG21 nanoformulation cures *P. berghei* sporozoites (left panel), blood (middle panel) and liver stages, leading to 100% survival (right panel). This is with oral administration of a single dose of 2.5 mg/kg or 3 doses at 0.625 mg/kg. Single dose causal prophylaxis in 5 C57BL/6 albino mice at 2.5 mpk dosed on day 0, 1 h after intravenous administration of 10,000 *P. berghei*sporozoites. Shown is 3 dose causal prophylaxis treatment in 5 C57BL/6 albino mice at 0.625 mpk dosed on days −1, 0 and +1. Representative figure showing survival (right panel), luminescence (left panel) and parasitemia quantitated by flow cytometry (middle panel) for 5 mg/kg.” (**F**) Persisters of a RPS13 Λ strain of *Toxoplasma* can persist long times by exiting the cell cycle at G1 (**G**). The nanoformulation highly active *in vivo* against RH strain tachyzoites indicates that oral formulation in a conventional manner will likely be feasible. Biomarkers of illness, not shown, can also be a measure of efficacy in future studies, including circulating miRs, specific serum proteins and T2 weighted abnormalities in brain MRI. Part II. Vaccines on the horizon in pre-clinical studies. (**H**) Self assembling nanoparticle immunosense vaccine is potent in protection of mice (134, 137-9). RNA dendrimers also are promising in our vaccine work (Melo, Zhou, Weiss, Irvine, McLeod et al, In preparation, 2022). (**I**) Protection of primates eliminating death in French zoos with porous nanoparticles loaded with *T.gondii* lysate administered intranasally [https://www.inrae.fr/actualites/vaccine-contr-toxoplasmose-singes-saimiris]. Additional work of of others is referenced as well [100–135]. (**J**) Importance of screening and treating during gestation for those who are seronegative, new medicines and vaccines are emphasized in the words and image of J. Morel and her familly. Other images and Figure legend for Part I are reproduced with permission from Frontiers Cell Infect Microbiol, Nature Partners Journal Vaccines, and from Vaxinano

Recurrent disease demonstrates the great need for new and improved medicines which eliminate the dormant form of the parasite and vaccines to prevent acquisition in the first place. There is recent progress in these areas of research (Table 5 and Fig. 5) ( 64, 84- 135) with the hope that there will be resources that enable some of these approaches to treatment and prevention to reach the clinic resulting in definitive cure and prevention by vaccines. In animal models this appears to be feasible with very prompt treatment [84••, 85••, 86••]. One recent study suggests that there could be less new, recurrent retinal disease with prompt treatment of the acute acquired infection, at least in Colombia, and that a detectable allelic variation in a secreted Rhoptry protein, Rhoptry Protein 16 (ROP 16) is the responsible virulence factor at least in Colombia [97].

#### Discussion

Overall, in the United States, treatment algorithms for prenatal and postnatal care evolved in parallel with models for care from Europe and some South American countries. This experience, clinical studies and randomized control trial provided substantial insight into pathogenesis and presentation of toxoplasmosis [1••, 2, 3••, 4••, 5••, 6••, 7••, 8••, 9••, 10••, 11••, 12, 13, 14••, 15••, 16••, 17••, 18•, 19•, 20•, 21•, 22•, 23, 24••, 25, 26, 27•, 28•, 29–30, 31•, 32•, 33••, 34•, 35•, 36, 37•, 38•, 39••, 40•, 41•, 42••, 43••, 44••, 45, 46•, 47••, 48••, 49, 50•, 51•, 52, 53••, 54, 55••, 56••, 57•, 58••, 59•, 60••, 61, 62••, 63, 64.••••, 65, 66••, 67••, 68••, 69, 70, 71.••••, 72••, 73••, 74••, 75••]. This information, for the NCCCTS in the United States, was summarized in Part I Tables 1, 2, 3, 4, 5. Difficulties in obtaining optimal care was made more apparent by the pandemic and are highlighted herein. The difficulties with medicine access are in part related to cost and limited distribution and are summarized in Part I Tables 4 and 5. Guidelines for care in the United States are also presented in Part I.

In Panamá’, with respect to medical infrastructure and protocols, we started by creating a legal framework and an evidence-based clinical model for a preventive care approach to congenital toxoplasmosis; we then studied the national levels of compliance with these efforts. In October 2014, Decreto ejecutivo No. 1617 was passed in response to a study conducted by Hospital del Niño researchers at Hospital Santo Tomás; this study suggested seroprevalence of toxoplasmosis in Ciudad de Panama could be as high as 50 percent. The law passed in 2014 mandated that in Panamá, pregnant women be tested for congenital toxoplasmosis twice during gestation and there should be reporting of cases of infection to the Ministry of Health. However, our spatial analysis studies in 2016 and 2017 found that, even over two years after the law went into effect, screening rates varied from 0% to 60% regionally and were at about 39% nationwide. These findings led to investigations of why some areas are more compliant, as well as how these differences in compliance could inform educational approaches. Our later larger aggregate studies on *Toxoplasma*’s prevalence in Panamá—one of which used standard-of-care serologic testing for about 3500 pregnant women—enhanced national awareness of congenital toxoplasmosis and provided evidence for the considerable benefits of testing pregnant women consistently for seroconversion. In the meantime, as suggested by the low overall national screening rate, children with severe disease due to congenital toxoplasmosis are not uncommon in Panamá.

While increasing compliance is important, we also found that Panama could also benefit by implementing a more rigorous screening protocol than what Decreto ejecutivo No. 1617 stipulates. This is because—as our 2016 and 2017 studies showed—a *Toxoplasma* infection occurring between the two stipulated tests could remain undetected for a significant period of time. While 23 women were classified as IgG-/IgM- prior to these studies, we found in a second screening near the end of gestation that they had become IgG+, IgM-. We do not know whether these patients had false negative tests at first, or if their serologies missed the time when tests for acute infection were positive, suggesting that months had occurred after seroconversion. This means that these women could have been acutely infected at some point earlier in their gestation and thus could have transmitted the infection to their fetus when preventive therapy could have been implemented. These findings re-emphasize that—while Decreto ejecutivo No. 1617 is a good start—the optimal conditions for rapid diagnosis, prompt treatment and accurate tracking for seroconversion cannot depend only on two widely spaced screenings. Instead, studies in France, Austria and Colombia have suggested that identifying seronegative (and at-risk) mothers prior to gestation and then testing these women monthly for anti-*Toxoplasma* antibodies is a very effective and relatively cost-saving model for ensuring the fewest congenital toxoplasmosis -related adverse outcomes for infants [43••, 44••, 51•]. A longer delay between accurate diagnosis and appropriate treatment increases the likelihood that a congenitally infected child develops more severe illness or remains undiagnosed. As such, our study provides useful data for the Panamanian Ministry of Health about this problem and an opportunity to intervene. Even if there were universal compliance with Decreto ejecutivo No. 1617, testing women twice during gestation should be considered merely one early step toward a more comprehensive national gestational screening program.

With this ideal screening program in mind, we are hoping to implement POC tests for anti-*Toxoplasma* antibodies in specific practices in Panamá. We have already had some success with the LDBIO *Toxoplasma* ICT IgG-IgM test (LDBIO Diagnostic, Lyon, France), a test that meets World Health Organization’s ASSURED (Affordable, Sensitive, Specific, User-friendly, Rapid, Robust, Equipment-free, Deliverable) criteria [43••, 44••]. The high performance of this test, as documented in previous studies, made it appealing to pilot its use in early studies in Panama in 2019. Since then, the LDBIO device has made it possible to know when false positive results were generated by Roche IgG and IgM or other commercial tests. The LdBio performed relatively well on 100 sera from pregnant patients in Panamá. Since a positive test in a screening program will be confirmed by a second test that should not interfere with its use. All the negative test values were negative in both Roche and LdBio POC tests. This test was CE marked in Europe and an extension of CE approval including whole blood use was done in December 2020. If there is approval for use in the United States by the FDA, it could build the momentum to use the LDBIO *Toxoplasma* ICT IgG-IgM device on a larger scale in serologic screening programs during gestation in multiple countries, including in Panamá, the United States and Colombia. There are ongoing larger scale studies in Colombia and in Morocco in progress now and plans to begin other larger scale studies in the United States soon.

Our studies on seroprevalence and testing (discussed in more detail below) generated discussion at Hospital Santo Tomás and Hospital del Niño regarding the possibility of creating a more comprehensive congenital toxoplasmosis screening program. Researchers and physicians from both institutions recognized the considerable spillover benefit in having regular care with monthly screening that used a test like the LDBIO device. To make certain that there was an organized system to ensure high quality health care, Hospital del Niño founded a perinatal infectious disease outpatient program to support diagnosis and care for affected infants and children. Although Hospital Santo Tomás is now ready to implement a monthly screening program using the POC test, this was put on hold due to the COVID-19 pandemic. In the meantime, Hospital Santo Tomás’s program provides access to previously unavailable or difficult to obtain medicines for patients with *Toxoplasma* infection, and physicians in this program also follow the disease course of congenitally infected children. A proposal to assure availability and easy, prompt access to low-cost, POC testing is being developed currently for all of Panamá. The current and upcoming programs at Hospital Santo Tomás and Hospital del Niño offer a paradigm for improving this aspect of healthcare infrastructure in Panamá.

In addition, recent cost-benefit analyses of congenital toxoplasmosis screening in the United States and Austrian contexts should contribute to consideration of expanding these programs throughout the country [1••, 2, 3••, 4••, 5••, 6••, 7••, 8••, 9••, 10••, 11••, 12, 13, 14••, 15••, 16••, 17••, 18•, 19•, 20•, 21•, 22•, 23, 24••, 25, 26, 27•, 28•, 29–30, 31•, 32•, 33••, 34•, 35•, 36, 37•, 38•, 39••, 40•, 41•, 42••, 43••, 44••, 45, 46•, 47••, 48••, 49, 50•, 51•, 52, 53••, 54, 55••, 56••, 57•, 58••, 59•, 60••]. The value of addressing access to reliable rapid on-site diagnostic testing with spillover benefit was discussed in detail in Begeman et al [43••]. Herein we present new work to build such a program in the United States, Panamá, Colombia and enhance that in France and other countries. This is part of a toolbox of approaches to attempt to eliminate toxoplasmosis.

Not only is there potential from the work itself, but participating in the process of generating these materials has also proven useful for each group of students that has taken a part in our work—including the public health students who addressed how NGOs and MINSA could work together to initiate toxoplasmosis care in the Embera indigenous communities of eastern Panama (see Fig. 3 and Supplement: Dhamsania et al.), both in the countries and also for the United States’ students who were graciously hosted in Panama and Colombia. This work began with the work of the NCCCTS in 1981 with very brave families with afflicted children with congenital toxoplasmosis brave enough to try a new treatment, demonstration of efficacy and safety through a phase 1 clinical trial and then a randomized controlled trial of two doses of medicines [1••, 2, 3••, 4••, 5••, 6••, 7••, 8••, 9••, 10••, 11••, 12, 13, 14••, 15••, 16••, 17••, 18•, 19•, 20•, 21•, 22•, 23, 24••, 25, 26, 27•, 28•, 29–30, 31•, 32•, 33••, 34•, 35•, 36, 37•, 38•, 39••, 40•, 41•, 42••, 43••, 44••, 45, 46•, 47••, 48••, 49, 50•] transitioning into a longitudinal study of outcomes and biology of the infection and a reference center for care. Noting the extremely favorable outcomes for those treated *in utero*, an attempt was made to develop a program for screening in the United States [61]. This includes building supportive infrastructure and addressing cost benefit and developing ways to implement screening at low cost with very high-quality tests, a program has grown in the United States. This has moved forward in France, Austria and Slovenia, developed at scale in Morocco moving toward screening programs including 2,000 persons. In Colombia screening has been implemented and Panama is contemplating this as well.

Novel anti-parasitic treatments and vaccines and studies of pathogenesis informing understanding of consequences of this infection are also ongoing in the US, Europe, Colombia and Brazil among other countries [1••, 2, 3••, 4••, 5••, 6••, 7••, 8••, 9••, 10••, 11••, 12, 13, 14••, 15••, 16••, 17••, 18•, 19•, 20•, 21•, 22•, 23, 24••, 25, 26, 27•, 28•, 29–30, 31•, 32•, 33••, 34•, 35•, 36, 37•, 38•, 39••, 40•, 41•, 42••, 43••, 44••, 45, 46•, 47••, 48••, 49, 50•, 51•, 52, 53••, 54, 55••, 56••, 57•, 58••, 59•, 60••, 61, 62••, 63, 64.••••, 65, 66••, 67••, 68••, 69, 70, 71.••••, 72••, 73••, 74••, 75••, 76–78, 79••, 80••, 81••, 82, 83, 84••, 85••, 86••, 87••, 88••, 89••, 90, 91, 92••, 93, 94, 95, 96, 97, 98••, 99••, 100, 101••, 102••, 103••, 104••, 105••, 106••, 107••, 108••, 109••, 110, 111, 112, 113••, 114••, 115••, 116••, 117••, 118••, 119••, 120, 121, 122••, 123••, 124, 125••, 126••, 127••, 128••, 129••, 130••, 131••, 132, 133••, 134••, 135, 136••, 137••, 138••, 139••]. Some of this is summarized by McLeod et al in Weiss and Kim (Editors) [64.••••] very recently with some studies referenced herein [1••, 2, 3••, 4••, 5••, 6••, 7••, 8••, 9••, 10••, 11••, 12, 13, 14••, 15••, 16••, 17••, 18•, 19•, 20•, 21•, 22•, 23, 24••, 25, 26, 27•, 28•, 29–30, 31•, 32•, 33••, 34•, 35•, 36, 37•, 38•, 39••, 40•, 41•, 42••, 43••, 44••, 45, 46•, 47••, 48••, 49, 50•, 51•, 52, 53••, 54, 55••, 56••, 57•, 58••, 59•, 60••, 61, 62••, 63, 64.••••, 65, 66••, 67••, 68••, 69, 70, 71.••••, 72••, 73••, 74••, 75••, 76–78, 79••, 80••, 81••, 82, 83, 84••, 85••, 86••, 87••, 88••, 89••, 90, 91, 92••, 93, 94, 95, 96, 97, 98••, 99••, 100, 101••, 102••, 103••, 104••, 105••, 106••, 107••, 108••, 109••, 110, 111, 112, 113••, 114••, 115••, 116••, 117••, 118••, 119••, 120, 121, 122••, 123••, 124, 125••, 126••, 127••, 128••, 129••, 130••, 131••, 132, 133••, 134••, 135, 136••, 137••, 138••, 139••]. There are novel inhibitory compounds that appear effective against both active rapidly and slow growing *Toxoplasma****.*** There are very promising small molecule inhibitors [84••, 85••, 86••], antisense [109], promising vaccines [98, 99, 114] and greater understanding of pathogenesis and consequences of infection [1••, 2, 3••, 4••, 5••, 6••, 7••, 8••, 9••, 10••, 11••, 12, 13, 14••, 15••, 16••, 17••, 18•, 19•, 20•, 21•, 22•, 23, 24••, 25, 26, 27•, 28•, 29–30, 31•, 32•, 33••, 34•, 35•, 36, 37•, 38•, 39••, 40•, 41•, 42••, 43••, 44••, 45, 46•, 47••, 48••, 49, 50•, 51•, 52, 53••, 54, 55••, 56••, 57•, 58••, 59•, 60••, 61, 62••, 63, 64.••••, 65, 66••, 67••, 68••, 69, 70, 71.••••, 72••, 73••, 74••, 75••, 76–78, 79••, 80••, 81••, 82, 83, 84••, 85••, 86••, 87••, 88••, 89••, 90, 91, 92••, 93, 94, 95, 96, 97, 98••, 99••, 100, 101••, 102••, 103••, 104••, 105••, 106••, 107••, 108••, 109••, 110, 111, 112, 113••, 114••, 115••, 116••, 117••, 118••, 119••, 120, 121, 122••, 123••, 124, 125••, 126••, 127••, 128••, 129••, 130••, 131••, 132, 133••, 134••, 135, 136••, 137••]. This progress in these areas will contribute to eliminating toxoplasmosis as a human disease in the future.

This work has become part of a global initiative to save the lives, sight and cognition of fetuses, infants and children [48••]. It has also provided a framework to study the effect of this parasite in causing neurodegenerative diseases, epilepsy and other diseases later in life as this parasite remains in the brain of more than 2 billion people lifelong [138]. New compounds with potential for cure [84••, 85••, 86••] and approaches for development for vaccines to prevent this disease [84••, 98, 99, 101••, 102••, 103••, 104••, 120, 121, 132, 133••, 134••, 135, 136••, 137••, 138••, 139••] are also underway.

They are inspired by the observations of afflicted children in these programs and the needs to build strong programs to assist in their care and prevention of this disease, and discovery and development of novel medicines and vaccines to treat and prevent it even more effectively.

## Supplementary Information

ESM 1 (PDF 1.01 MB)

ESM 2 (PDF 498 kb)
