# Supplementary material for: Building Programs to Eradicate Toxoplasmosis Part IV: Understanding and Development of Public Health Strategies and Advances “Take a Village”
Source: Curr Pediatr Rep. 2022 Aug 16;10(3):125–54. doi: 10.1007/s40124-022-00268-x (PMC9379243; doi:10.1007/s40124-022-00268-x)
Supplement: Supplementary file 1 — (PDF 1.01 MB) [file 40124_2022_268_MOESM1_ESM.pdf]

Building Programs to Eradicate Toxoplasmosis Part IV: Public Health

Mariangela Soberón Felín, JD,[1*] Kanix Wang, PhD,[2*] Andrew Grose,[3*] Zuleima Caballero, PhD,[4*] Ximena Norero, MD,[5*] Dora Estripeaut, MD,[5*] David Ellis, MD,[5] Catalina Raggi,[6,7] Catherine Castro, MD,[3,6*] Davina Moossazadeh,[6-8*] Margarita Ramirez,[6,7,9*] Abhinav Pandey,[6,7,9*] Aliya Moreira,[6,7,9*] Kevin Ashi,[3] Samantha Dovgin,[6,7] Ashtyn Dixon,[6*] Xuan Li, MD,[10*] Fatima Alibana Clouser, MD,[6] Ian Begeman,[3,6] Sharon Heichman, MD,[3,6*] Joseph Lykins, MD,[3,6] Delba Villavos,[4] Lorena Fabrega,[4] José Sanchez,[6,7] Connie Mendivil,[4] Mario L. Quijada, BSc.,[4] Silvia Fernández-Pirla,[1,11] Digna Wong, MD,[4] Mayrenis Ladrón de Guevara, MD,[4] Carlos Flores,[12], Jovanna Borace,[12] Anabel García, DVM,[4] Natividad Caballero, MD,[13] Claudia Rengifo-Herrera, DVM,[14]Stephanie Ross,[10] Mimansa Dogra,[6] Vishan Dhamsania, MD,[3,15] Nicholas Graves, MD,[3,15] Marci Kirchberg, MS,[15,16] Kopal Mathur, MS,[15,16] Ashley Aue,[16] Arturo Rebellon, MD,[17] Kenneth Boyer, MD,[10] Peter Heydemann, MD,[10] A. Gwendolyn Noble, MD, PhD,[18] Peter Rabiah, MD,[19] Shawn Withers,[6] Chunlei Su, PhD,[20] Daniel Vitor Vasconcuelos Santos, MD,[21] Alcibiades Villareal,[4] Ambar Perez,[4] Carlos Andrés Naranjo Galvis,[22] Mónica Vargas,[23] Laura Lorena Lopez,[23] Guillermo Padrieu,[24] Alejandra de la Torre, MD, PhD,[25] Daniel Nicolae PhD,[8] Andrey Rzhetsky, PhD,[2] Eileen Stillwaggon, PhD,[26] Larry Sawers, PhD,[27] Francois Peyon, MD, PhD,[28] Eduaordo Ortega-Barria MD[29], MichaelGrigg PhD[30], Gabrielle Britton, PhD,[4a] Jorge Motta, MD, MPH,[29,a] Jorge Gómez-Marín, MD, PhD,[23] Jagantha Rao Kosagishara, PhD,[4] Xavier Sáez Llorenz, MD,[5+a] Osvaldo Reyes, MD[12+a], Rima McLeod, MD, FACP, FIDSA, FAAAS[1-3,6,31,32+]

[1] Toxoplasmosis Programs and Initiatives in Panama, Ciudad de Panamá, Panama
[2] Institute for Genomics and Systems Biology, The University of Chicago, Chicago, Illinois
[3] Pritzker School of Medicine, The University of Chicago, Chicago, Illinois
[4] Instituto de Investigaciones Científicas y Servicios de Alta Tecnología AIP (INDICASAT-AIP), Ciudad de Panamá, Panama
[5] Department of Pediatrics Infectious Diseases/Department of Neonatology, Hospital del Niño, Ciudad de Panamá, Panama
[6] Department of Ophthalmology and Visual Sciences, The University of Chicago, Chicago, Illinois
[7] The College, The University of Chicago, Chicago, Illinois
[8] Department of Statistics, The University of Chicago, Chicago, Illinois
[9] The Global Health Center, The University of Chicago, Chicago, Illinois
[10] Rush University Medical School/Rush University Medical Center, Chicago, Illinois
[11] Academia Interamericana de Panamá, Ciudad de Panamá, Panama
[12] Hospital Santo Tomás, Ciudad de Panamá, Panama
[13] Hospital San Miguel Arcángel, Ciudad de Panamá, Panama

[14] Universidad de Panamá, Ciudad de Panamá, Panama
[15] Capstone Program Global Health Center, The University of Chicago, Chicago, Illinois
[16] Harris School of Public Policy, The University of Chicago, Chicago, Illinois
[17] Sanofi Aventis de Panamá S.A., Ciudad de Panamá, Panama
[18] Northwestern University Feinberg School of Medicine, Chicago, Illinois
[19] NorthShore Evanston Hospital, Evanston, Illinois
[20] Department of Microbiology, The University of Tennessee, Knoxville, Tennessee
[21] Universidade Federal de Minas Gerais, Minas Gerais, Brazil
[22] Universidad Autónoma de Manizales, Manizales, Colombia
[23] Universidad del Quindío, Armenia, Colombia
[24] The University of South Florida College of Public Health, Tampa, Florida
[25] Grupo de Investigación en Neurociencias, Universidad del Rosario, Bogotá, Colombia
[26] Department of Economics, Gettysburg College, Gettysburg, Pennsylvania
[27] Department of Economics, American University, Washington, D.C.
[28] Institut des agents infectieux, Hôpital de la Croix-Rousse, Lyon, France
[29] Secretaría Nacional de Ciencia, Tecnología e Innovación (SENACYT), Ciudad de Panamá, Panama
a Sistema National de Investigadores de Panama(SNI), Clyton,Panama
[30] Moleculr Parasitology Section Laboratory of Parasitic Diseases,National Institute s of Health,NIAIDmBethesdam MD.USA
[31] Toxoplasmosis Center, The University of Chicago, Chicago, Illinois
[32] Department of Pediatrics (Infectious Diseases), The University of Chicago, Chicago, Illinois

[+]To Whom Correspondence Should be Addressed

rmcleod@uchicago.edu

xsaezll@cwpanama.net

oreyespanama@yahoo.es

jrao@indicasat.org.pa

Supplement: compilation of contributors' original research, writing, and presentations

Authors' note: Most of the materials in this supplement come from independent investigations conducted by students who were affiliated with global health research programs at the University of Chicago. As more student contributors were graciously invited to work with individuals and institutions in Panama and Colombia, the research that these students completed and presented became part of a truly international public health initiative, one that quickly involved more institutions and collaborators than many of us had originally conceived. None of these projects would have been possible without the collaboration of numerous U.S. and in-country partners. As such, each contributor's principal partners are highlighted in the title page of each section in this supplement.

Table of contents:

DHAMSANIA ET AL. 1
Developing a Framework for Accessing Panama's Indigenous Communities 2

THE UNIVERSITY OF CHICAGO
Harris Public Policy

Vishan Dhamsania, Nick Graves, Marci Kirchberg, and Kopal Mathur
with Mariángela Soberón Felín, JD; James McAuley, MD, MPH; Rima McLeod, MD

“Developing a Framework for Accessing Panama’s Indigenous Communities: Forming Relationships to Advance Toxoplasmosis Screening”

Case Mentor: Dr. Rima McLeod

Group:

- Vishan Dhamsania
- Nick Graves
- Marci Kirchberg
- Kopal Mathur

PROBLEM DESCRIPTION

Disease background

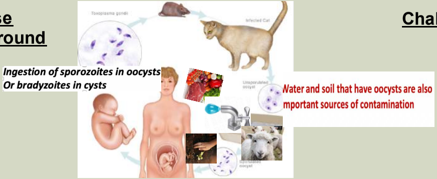

- Cause: Parasite Toxoplasma Gondii; mother to fetus
- Effects: Significant neurologic and ophthalmologic damage
- Sero-prevalence: Estimated 50% in Panamanian women of childbearing age
- Screening: Available but concentrated in Panama City (80% of population); Inconsistent rates

Challenges

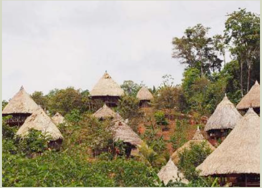

- Expansion of outreach interventions to the rural areas and certain communities
- Limited access to public healthcare systems by the indigenous population
- Resulting in poor health outcomes - infant mortality 3x higher than the national rate

TARGET POPULATION: EMBERA INDIGENOUS GROUP

- Size: Total indigenous population of 194,166 (11.8% of total Panamanians); out of which 35,000 Embera population

- Location: Spread across country, but established in Darien (~15,000 still living there)

- Characteristics:
  - Organized into small social groups
  - Noko= chief, committees on health and "women's issues"
  - Unique language, many are bilingual
  - No formal "religion"
  - Traditional medicine, private life, importance of having space respected
  - Slash/burn agriculture, live in open air huts

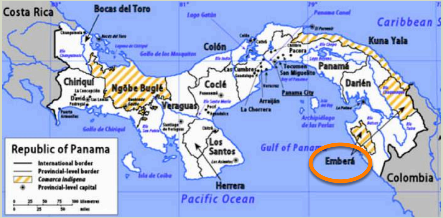

SCOPE

We seek to address the issue of gaining access to the indigenous population of Panama, with the long term goal of providing more consistent prenatal care to include screening for Toxoplasmosis

Context

- Create guidelines on “how to develop relationships with indigenous groups”
- Propose new interventions or use of existing ones
- Explore public vs. private sector partnerships

Environment

- Perspectives at individual, community and country level
- Partner with existing groups or health workers

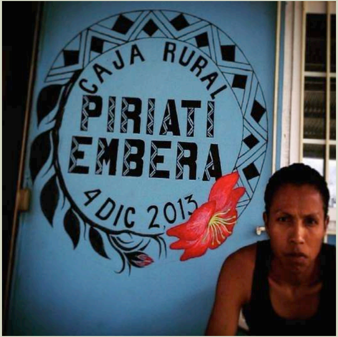

POSSIBLE APPROACHES

Access via Government (MINSA)

Use government programs and resources to deliver healthcare to Embera and integrate health activities

Access via NGOs

Partner with existing NGOs to build relationships and gain access to the communities

EVALUATION CRITERIA

FACTORS

- Political
- Economic
- Cultural
- Sustainable

CRITERIA

- Red diamond: Disadvantage
- Yellow diamond: Unequivocal
- Green diamond: Definite advantage

POLITICAL

	MINSA	NGO
**Political Viability (e.g. need & political will)**		
**Legal Support & Statutes**		
**Areas of Conflict (e.g. disputes between parties)**		

Salud
Ministerio de Salud
Panamá

	MINSA	NGO
**Human resources**	◆ (red)	◆ (yellow)
**Costs and overall funding security**	◆ (green)	◆ (yellow)
**Economic benefits for Embera**	◆ (yellow)	◆ (green)

ECONOMIC

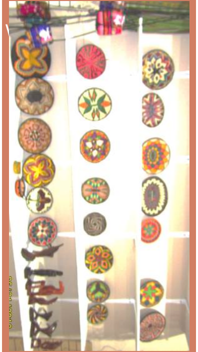

	MINSA	NGO
**Cultural Norms**	Red diamond	Yellow diamond
**Language**	Yellow diamond	Green diamond
**Community Involvement**	Red diamond	Yellow diamond

CULTURAL

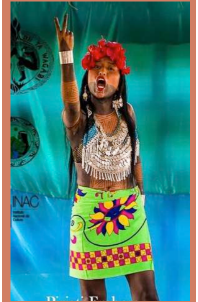

SUSTAIN-ABILITY

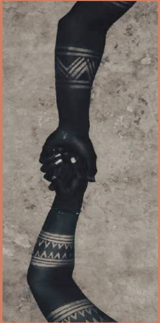

	MINSA	NGO
Capacity Building & Continuous Training	◆ (green)	◆ (yellow)
Monitoring, Evaluation and Feedback Mechanism	◆ (yellow)	◆ (red)
Incentives	◆ (green)	◆ (green)

PUTTING THE CRITERIA TOGETHER

	MINSA	NGO	Both
**Political**	◆ (green)	◆ (red)	◆ (green)
**Economic**	◆ (yellow)	◆ (green)	◆ (green)
**Cultural**	◆ (yellow)	◆ (yellow)	◆ (green)
**Sustainability**	◆ (green)	◆ (yellow)	◆ (green)

PARTNERSHIP OFFERINGS

MINSA

- Stable funding
- Long term goals
- Language requirements, capabilities
- Capacity building of NGO workers
- Robust overall M&E
- HR & other available resources

NGOs

- Will & manpower
- Flexibility
- Capacity building of community workers
- Implementation & Service delivery

STEPS FOR ASSESSMENT

Country level assessment

- Multi-disciplinary approach: infrastructure, HR, planning methods, needs and gaps

Community level assessment

- Identify target groups, specific health & social needs, available resources, modes of access

Individual level assessment

- Identify health workers, community mobilizers, regional and religious leaders, health educators

International practices

- Identify similar programs for indigenous & tribal communities in different countries and adapt

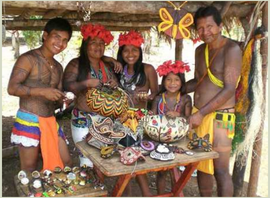

WHO TO CONTACT & WHAT TO ASK?

**Federal government**	• MINSA branches: Funding, structure
**Donor organizations**	• Current funding allocations and future opportunities
**Medical schools**	• Faculty: Research, community engagement and partnerships
**NGOs**	• Ciudad del Saber: Reach and programs
**Local hospitals**	• Hospital administrators: Outreach
**Community health workers**	• Perspectives and challenges
**Local government**	• Cacique: Funding, structure, receptiveness, culture and roles

LIMITATIONS

- Time limitations made it difficult to obtain primary data
- Little in country experience or community exposure
- Subjective quality of evaluation
- Limited criteria
- Changes in administration and administration priorities
- No current models or data for cost of operations
- Gap between policies and implementation
- Personal relationships are of critical importance

Immediate Recommendations

If a researcher were going to Panama this summer:

1. **Introduction and meeting with MINSA, specifically Dirección de Asuntos Sanitarios Indígenas and Commission Nacional de Medicina Tradicional**
2. **Exploration of Ciudad del Saber entities and identification of NGOs currently working with the Embera**
3. **Develop a health beliefs and attitudes survey and disseminate to Embera via aforementioned organizations.**

Questions?
