# Supplementary material for: Building Programs to Eradicate Toxoplasmosis Part IV: Understanding and Development of Public Health Strategies and Advances “Take a Village”
Source: Curr Pediatr Rep. 2022 Aug 16;10(3):125–54. doi: 10.1007/s40124-022-00268-x (PMC9379243; doi:10.1007/s40124-022-00268-x)
Supplement: Supplementary file 2 — (PDF 498 kb) [file 40124_2022_268_MOESM2_ESM.pdf]

Supplement Part B for Part IV

[illegible]

A Mother's Testimony in Support of The Prenatal and Neonatal Congenital Toxoplasmosis Prevention and Treatment Act

Posted on July 24, 2014 by John Easton

This article is a supplement to our interview with toxoplasmois expert Rima McLeod, MD, on the impact of having nearly 2 billion people in the world infected with the parasite that causes the disease. You can read the original article here.

Dana Morel suffers from toxoplasmosis. Janet, her mother, testified before the Illinois State Senate accompanied by Rob and Dana, in support of Senate Bill 3667, the proposed Prenatal and Neonatal Congenital Toxoplasmosis Prevention and Treatment Act. The bill provides that health care professionals "shall provide counseling for Toxoplasmosis and testing for T. gondii when providing care to a pregnant woman during her pregnancy." An excerpt from her testimony is below.

It has not yet become law. Practice guidelines are being formulated by the Infectious Diseases Society of America by a Committee chaired by McLeod. Guidelines concerning management of congenital toxoplasmosis are just now being published by McLeod et al in Current Pediatrics Reports and in An Atlas of Neonatology, in press, which are expected to become a foundation for legislation proposed in the future. A cost-benefit analysis has indicatec that gestational screening programs are likely to be cost effective in the United States (Stillwaggon et al, Plos Neglected Tropical Diseases, 2011).

"Imagine with me a young mother happily awaiting the arrival of her first child. She does everything she knows to secure the baby's health. And then everything goes wrong.

Good morning, ladies and gentlemen. My family and I are here to show you the reality of what Toxoplasmosis can do if it goes undetected and untreated. We offer our story in hopes that you will pass Senate bill number 3667, doing all you can to prevent the overwhelming effects of this diseas on citizens in your care.

I contracted Toxoplasmosis in my first trimester. I will never know how. The doctor did not educate me or test me for it. I was not given the opportunit to prevent the devastation this disease would have on my baby. It had a lot of time to go unchecked and do a lot of damage.

I know one of the hesitations about passing Senate Bill 3667 is that we, the people, would be dictating how obstetricians conduct the practice of medicine. Don't doctors have a sworn duty to protect the lives and health of their patients? Our family and many, many others like us, are proof that not all doctors are currently fulfilling their duty.

My daughter Dana was born six week premature with severe damage to her brain and her eyes. She is now 22 years old. She has very little cortex to he brain. She has cerebral palsy. She is blind. She has severe developmental delays. Dana requires our full care. She cannot sit unassisted, walk, talk, feed herself, dress herself or toilet herself. My husband and I still change her diapers day and night.

The toll on her body has been tremendous. She has had ten surgeries, two of which were so traumatic we almost lost her. She has a tube that drains excess fluid from her brain. She has a metal rod in her back to help straighten severe curvature to her spine so her organs and lungs can continue to function. She has a pump surgically inserted to give her constant doses of a medicine to help relax her muscles, from further contorting her body, whicl by the way, is only minimally successful. One hip was reconstructed; the other is currently dislocated.

I'm aware that another concern about this bill is the possible cost. We have been adding up the costs of Dan's treatment and care. Remember, I could have been tested for about $12. Just the shunt surgery alone cost $100,000. Dana has had four. Her back and pump surgeries were much more. Then there are special education expenses, physical therapy, occupational therapy, speech therapy, music therapy, special equipment such as wheelchairs, bath chairs and orthotics, respite care, attendant care, habilitation, diapers and mediation. To refill her pump costs almost $7000 every six weeks. For Dana's care alone we are now into the millions. That is absolutely astronomical. And I could have had a $12 test.

I hate what toxoplasmosis has done to my daughter. It has stolen the dreams I once had for her, and from me. I cannot work full time because of the demands of her care. Our whole family has experienced emotional and financial strain. Yet through this all, we have loved this young lady. She is remarkably determined to endure. She laughs, she enjoys being a part of an active family life.

Dana has a younger sister Rebecca who is in college right now. I wish you could know her. She is one of the smartest, wittiest, kindest people I know. Life has been difficult for her, too. What bothers her most are the constant stares from disapproving or curious people. She is studying fine arts with a major in animation. Her work is influenced by her love and protective instincts for her sister. Watching Rebecca I can't help but think of what Dana life would have been like if her infection were caught early on.

In fact I was just talking with Dana about that the other day when I was driving her home from her adult day program. I mentioned that if it were not fo disabilities caused by toxoplasmosis, she would be probably be finishing her final year in college right now. I wondered out loud what she would be studying. … What would her future have held?
